# Hepatic macrophage mediated immune response in liver steatosis driven carcinogenesis

**DOI:** 10.3389/fonc.2022.958696

**Published:** 2022-10-05

**Authors:** Taojian Tu, Mario M. Alba, Aditi A. Datta, Handan Hong, Brittney Hua, Yunyi Jia, Jared Khan, Phillip Nguyen, Xiatoeng Niu, Pranav Pammidimukkala, Ielyzaveta Slarve, Qi Tang, Chenxi Xu, Yiren Zhou, Bangyan L. Stiles

**Affiliations:** ^1^ Pharmacology and Pharmaceutical Sciences, School of Pharmacy, University of Southern California, Los Angeles, CA, United States; ^2^ Department of Pathology, Keck School of Medicine, University of Southern California, Los Angeles, CA, United States

**Keywords:** macrophages, Kupffer Cells, steatosis, liver cancer, inflammation

## Abstract

Obesity confers an independent risk for carcinogenesis. Classically viewed as a genetic disease, owing to the discovery of tumor suppressors and oncogenes, genetic events alone are not sufficient to explain the progression and development of cancers. Tumor development is often associated with metabolic and immunological changes. In particular, obesity is found to significantly increase the mortality rate of liver cancer. As its role is not defined, a fundamental question is whether and how metabolic changes drive the development of cancer. In this review, we will dissect the current literature demonstrating that liver lipid dysfunction is a critical component driving the progression of cancer. We will discuss the involvement of inflammation in lipid dysfunction driven liver cancer development with a focus on the involvement of liver macrophages. We will first discuss the association of steatosis with liver cancer. This will be followed with a literature summary demonstrating the importance of inflammation and particularly macrophages in the progression of liver steatosis and highlighting the evidence that macrophages and macrophage produced inflammatory mediators are critical for liver cancer development. We will then discuss the specific inflammatory mediators and their roles in steatosis driven liver cancer development. Finally, we will summarize the molecular pattern (PAMP and DAMP) as well as lipid particle signals that are involved in the activation, infiltration and reprogramming of liver macrophages. We will also discuss some of the therapies that may interfere with lipid metabolism and also affect liver cancer development.

## Introduction

Metabolic disorders, particularly obesity increases the risk of a number of cancers, e.g. colon, mammary, pancreas, liver (1, 2), etc. Obesity, which occurs in half of the US population, is now recognized as a confounding factor for cancer-related death (3, 4). The contribution of lipid dysfunction to cancer is particularly high for liver cancer. The mortality risk for liver cancer is estimated to be 4.52-fold higher in men with >35 body mass index (BMI) compared with those with BMI <29 (1). Liver steatosis is a common comorbid disease for liver cancer and is associated with metabolic diseases including obesity, insulin resistance (IR), and diabetes as well as in other related disorders such as alcohol usage disorders (5). While hyperinsulinemia, hyperglycemia, and hyperlipidemia as a result of peripheral insulin resistance and metabolic disorder can directly contribute factors to promote tumorigenesis (6), the resulting development of liver steatosis due to these conditions directly establishes the microenvironment to promote tumor development. This review will focus on the local tumor microenvironment in liver steatosis for its role in promoting cancer development.

## Contribution of steatosis to liver cancer

In the liver, steatosis is defined when at least 5% of lipid droplets are accumulated among hepatocytes in the histopathological diagnosis (7, 8), and is classified as alcoholic or nonalcoholic forms due to etiology. Non-alcoholic fatty liver disease (NAFLD) and non-alcoholic steatohepatitis (NASH) develop in patients with metabolic syndromes including obesity, IR and diabetes (9, 10) whereas alcoholic liver disease (ALD) and ASH (alcoholic steatohepatitis) are caused by excessive alcohol drinking which also contributes to lipid metabolic dysfunction (11, 12). While simple fatty liver is reversible by lifestyle changes, ASH and NASH can progress to more morbid forms of liver pathologies including fibrosis/cirrhosis and is highly associated with liver cancer (13).

Patients with varying degrees of steatosis are susceptible to hepatocellular carcinoma (HCC) and cholangiocarcinoma (CCA), the two dominant forms of liver cancer. In particular, the NAFLD-HCC incidence ratio increased significantly (1.92-fold for men and 12.7-fold for women) in the last 20 years whereas it decreased or remain unchanged for many other major etiologies of HCC (14). This increase is concurrent with the increase of obesity epidemic particularly in women, suggesting a role of lipid dysfunction in liver carcinogenesis. Consistently, alcohol consumption and associated alcoholic liver disease was estimated to be an independent risk factor for poor disease-free survival, particularly in non-virus hepatitis associated HCC (15).

Earlier studies using chemical carcinogen to induce cancer formation found that high fat diet (HFD) feeding significantly induced cell proliferation in diethyl nitrosamine (DEN) induced HCC models (16). While this observation is supported by high fructose, high cholesterol and alcohol feeding studies (17–19), other experiments show that HFD protects against DEN induced liver injury, leading to reduced HCC (20, 21). Avoiding the complications of chemical induced injury, genetic models were used to explore liver cancer development. Liver cancer is highly heterogenous on the pathohistological levels as well as genetic landscape. In recent years, exome sequencing has led to the discovery of TERT, CTNNB1 and TP53 as the dominant mutations and PI3K/AKT/PTEN/mTOR together with MAPK pathway as the primary signaling pathways that promote liver cancer development together with Wnt/β-catenin signaling pathway (22, 23). Mutation of TERT1 promoter is found to be a primary characteristic of NAFLD associated liver cancer (24) and loss of telomerase promotes metabolic dysfunctions in hepatocytes (25). Activating mutation of CTNNB1 (encodes β-catenin) occurring in 37% of HCC is thought to support the growth and transformation of liver cancer stem cells (26–29). As such, activating mutation of CTNNB1 confers the oncogenic potential of β-catenin and promotes HCC development (26, 27). Interestingly, manipulation of neither TERT, CTNNB1 nor TP53 by themselves is sufficient to result in liver tumor development (25, 26, 30, 31). Activation of PI3K signaling pathway, however unequivocally resulted in the development of HCC and CCA. Activating PI3K/AKT signal *via* deletion of Pten showed spontaneous tumor development following steatosis and fibrosis (32–35). The PI3K/AKT signal upregulation results in increased lipid anabolic metabolism in addition to acting as a pro-growth and pro-survival signal (35–45). In the Pten deletion model, inhibiting steatosis attenuates or abolishes tumor development, suggesting that steatosis is required for liver tumor development (32, 33), whereas short term feeding of HFD accelerates the development of tumors (46). The PI3K/AKT signal is necessary for driving the steatosis phenotypes in the liver (35, 37). As such, introduction of activated AKT delivered through hydrodynamic injection of myristylated AKT is necessary to drive the development of HCC and CCA for a number of signals including Notch, YAP, Shp2, Hippo and others (28, 47–49). Consistent with this notion, combining other genetic models with non-genotoxic chemicals and diet manipulations demonstrated that liver injury and steatosis promotes the development of tumors (28, 31–33, 50–52). In several mouse models including those lacking p53 and Indian Hedgehog, consumption of a Western-style diet, or a high-fat/high-cholesterol diet to the point of developing hepatic steatosis was shown to promote higher liver tumor incidence than the control diet group (53, 54).

**Figure 1:**
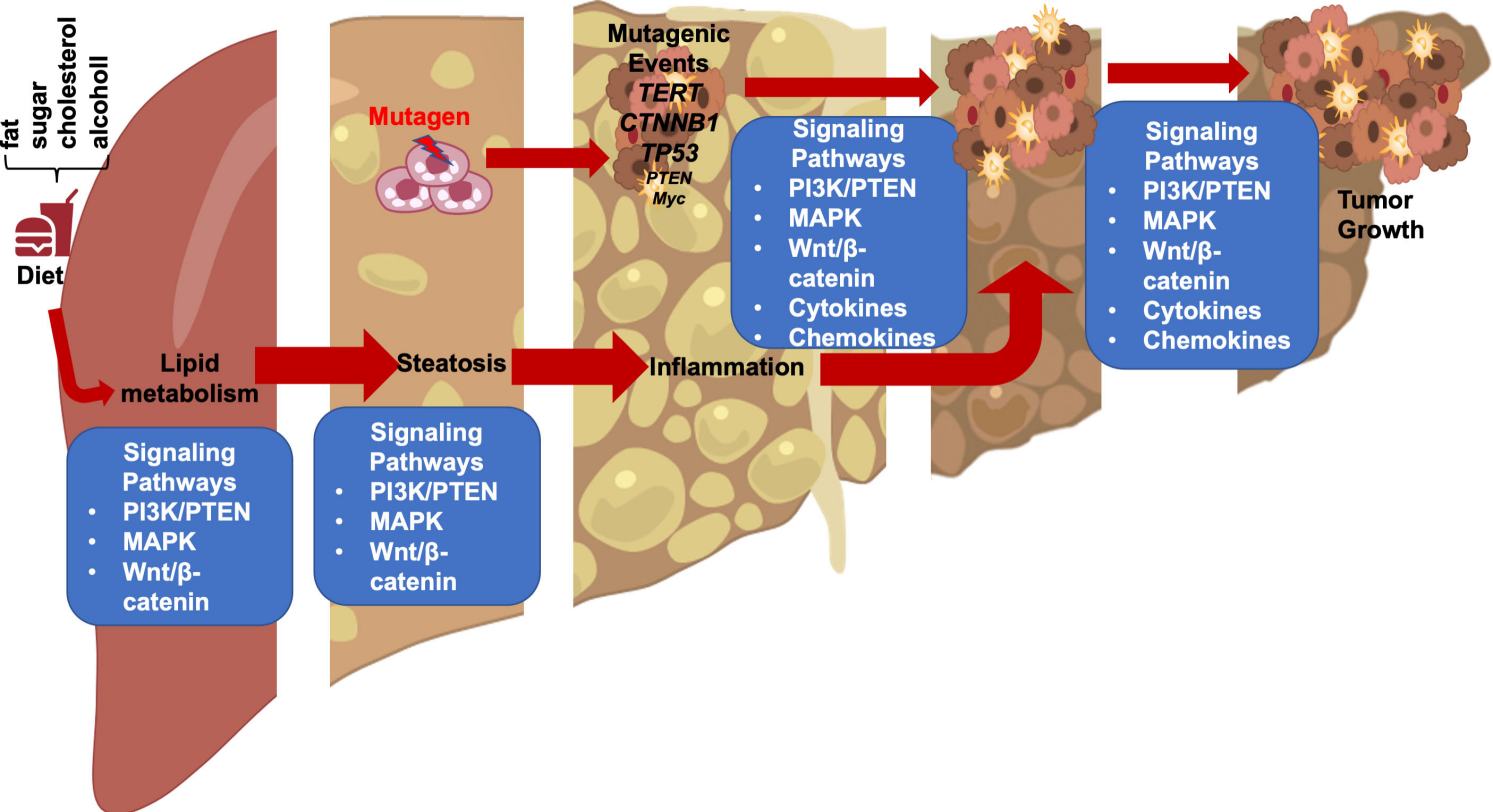
Steatotic Liver Damage Establishes a Tumor Microenvironment. The primary functions of the liver are metabolism and detoxication. Nutrients from the gut are metabolized in the liver involving the insulin regulated PI3K/PTEN pathway. Wnt/β-catenin signaling also plays important role in regulating the metabolic and detoxicating functions of the liver as it regulates liver structure and zonation. Following a diet containing high fat, sugar, cholesterol, or alcohol, activation of these signals results steatosis. The consequence cell death due to steatosis and loss of liver structure leads to inflammatory cell infiltration. Inflammatory mediators produced due to liver inflammation propagate any genotoxic events as the induce the proliferation of tumor initiating cells that carry mutations of *TERT, CTNNB1, TP53* and to a lesser extend *PTEN* and *MYC* as well as others. The Wnt/β-catenin, PI3K/PTEN and MAPK signaling pathways as well as cytokine and chemokine are all implicated in the proliferation of the tumor cells and play roles in propagating the initial mutagenic events.

In these genetic models where HFD feeding accelerates/promotes tumorigenesis, liver injury is a main consequence associated with steatosis (33, 46, 55). In fact, the effect of p53 on hepatocytes apoptosis may have contributed to the lack of tumorigenic effects observed in p53 deletion mice (31, 51) as p53 deficiency protected hepatocytes from undergoing apoptosis in response to HFD feeding and subsequent liver injury (56). Similarly, while activated β-catenin mutation is capable of promoting hepatocyte regeneration, the genotoxic effect requires steatosis and/or liver injury to promote liver cancer development (26, 28, 30). In fact, the function of β-catenin in sustaining normal hepatocyte function explains how β-catenin loss also promotes a protumor environment (57, 58). Deletion of β-catenin leads to loss of liver zonation, resulting in spontaneous repopulation of β-catenin+ cells due to hepatocyte death associated with loss of zonation. The death of hepatocytes also leads to cancer development from the β-catenin+ cells when genotoxic chemicals are introduced (29, 58, 59). Similar to chemical induced injury, steatotic injury has been shown to induce Wnt signal in the liver and elsewhere (60–63). Together, these studies suggest that liver steatosis establishes a microenvironment that promotes the growth of liver cancer cells and permits the expansion of any initial genotoxic events to develop into tumors (**Figure 1**).

## Macrophage response to steatotic liver injury: A double-edged sword in liver carcinogenesis

The liver is known as an immunosuppressive organ as illustrated by the lower dose of immunosuppressive therapy needed for liver transplantation as compared with other organ transplantations (64, 65). Liver macrophages play a critical role in this process. There are 2 basic populations of macrophages in the liver, the local proliferating Kupffer cells and the infiltrating monocyte derived macrophages (66). Kupffer cells are located within the liver sinusoids and play surveillance functions by monitoring pathogens coming into the liver. Being the largest tissue resident macrophage population in the body, Kupffer cells are the first responders in liver immune system. Unlike monocyte derived macrophages, Kupffer cells are highly effective in binding and clearing *Escherichia coli (E. coli)* brought in *via* the portal circulation (67).

**Figure 2:**
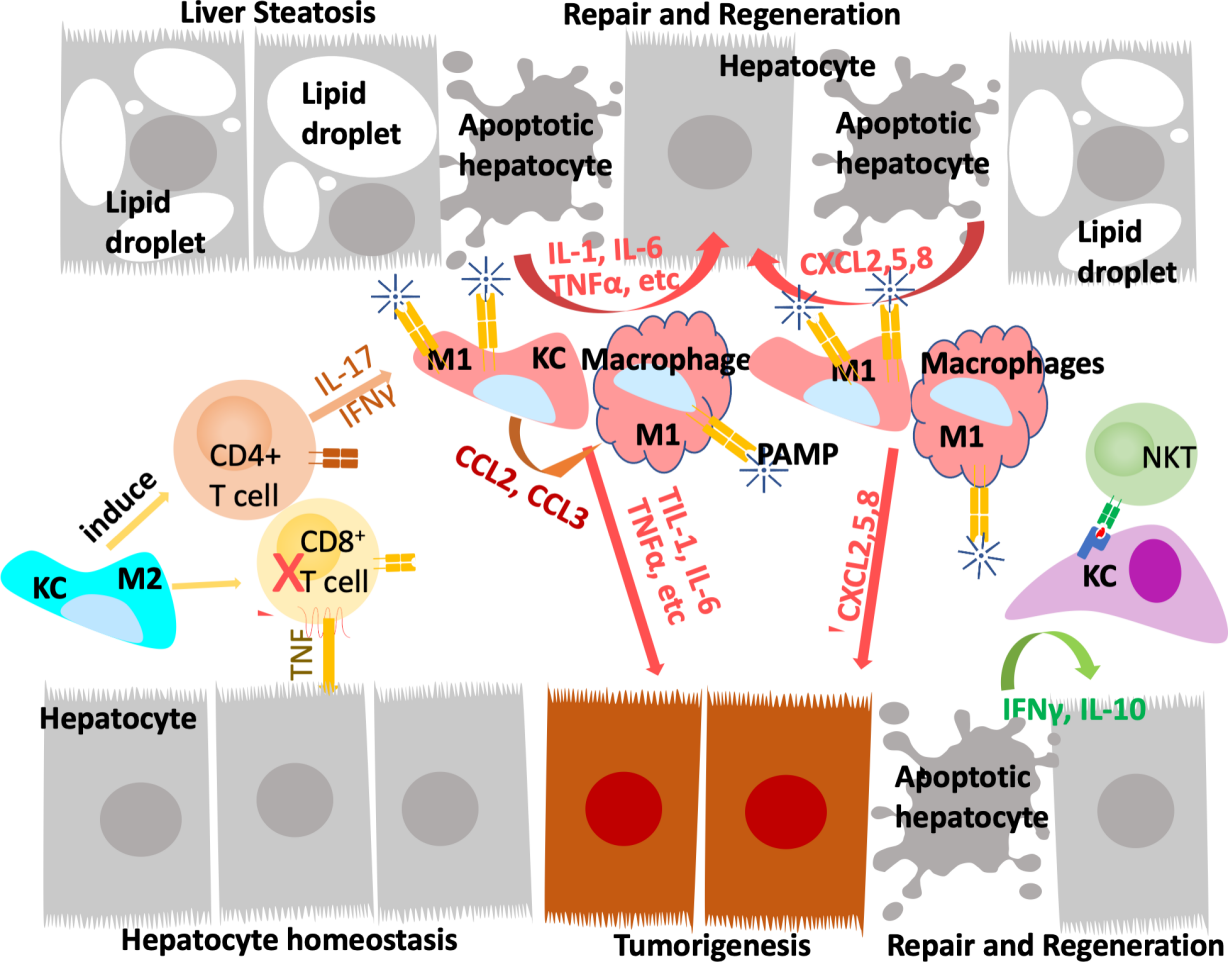
Innate immune system regulate liver repair and tumorigenesis due to steatosis. The liver developed a unique immune response system that tolerate gut bacterial-induced inflammation while eliminating them at the same time. During hepatocyte homeostasis (grey cells), Kupffer cells (KC) binds to and eliminate gut bacterial while producing anti-inflammatory cytokines to inhibit CD8+ cytotoxic T cells and induce their apoptosis. At the same time, Kupffer cells also induce antigen specific CD4+ Treg cells to assume tissue repair functions. In response to chronic injury presented in NAFLD and liver steatosis (grey cells with lipid droplets), Interleukine-17 (IL-17) and interferon g (IFNγ) produce from Tregs recruit monocyte derived macrophages as well as activating a M1 proinflammatory program in Kupffer cells. The proinflammatory cytokines produced by these M1 macrophages/Kupffer cells including IL-1, IL-6, TNFα, etc induces hepatocyte proliferation to repair the damaged tissues and replace apoptotic hepatocytes due to steatosis. These proinflammatory cytokines also establishes a pro-tumor microenvironment as they propagate any genotoxic events that are present in the tumor initiating cells (orange cells). The M1 macrophage/Kupffer cells also produces chemokines to promote hepatocyte and tumor cell proliferation, leading to tissue repair and/or tumorigenesis.

During homeostasis, Kupffer cells, but not monocyte derived macrophages, present antigens to induce immune tolerance through expansion of select regulatory T-cells and inhibition of T cytotoxic lymphocytes and to induce apoptosis in other T-cells (64, 68). In response to inflammation that cannot be cleared by Kupffer cells alone including those induced by pathogens and injury, inflammatory mediators released by Kupffer cells also recruit other inflammatory cells including monocytes-derived macrophages and neutrophils in addition to subsets of CD4+ and CD8+ T lymphocytes and NK/NKT cells (**Figure 2**). In particular, neutrophils, being the most abundant leukocytes in circulation are the first responders to acute inflammation to clear pathogens and damaged/dying cells. Similar to macrophages, neutrophils are highly enriched in the steatotic livers and depletion of neutrophils protects mice from experimentally induced steatohepatitis (69). Together, these infiltrating immune cells crosstalk with macrophages to clear pathogens and damaged tissues. In chronic injury conditions such as those presented by ALD and NAFLD, these inflammatory cells establish an environment that is pro-tissue repair and returning to homeostasis on the one hand; and pro-tumor growth when genotoxic events are present on the other. Recognition of damaged hepatocyte-released molecules by macrophages is important in the propagation of the signals and the sustained inflammatory response (see later section). Interaction of macrophages with other cell types such as cholangiocytes and hepatic stellate cells are also important in the disease progression and the establishment of the tumor microenvironment. This review focuses on the role of macrophages and macrophage produced inflammatory mediators. The interactions of macrophages with other inflammatory cells and their function in the tumor immune environment is also important for liver cancer development (70).

### Steatotic liver damage establishes a pro-inflammatory tissue microenvironment

Early studies showed that administration of liver toxicants such as carbon tetrachloride (CCL4) and 3,5-Diethoxycarbonyl-1,4-Dihydrocollidine (DDC) provoke the growth and infiltration of macrophages in the liver (71, 72). In patient samples, macrophages have been observed to be recruited to the NASH livers (73). These macrophages play roles in the disease progression of steatosis by producing inflammatory factors that sustain injury (6, 65, 74). In B6 mice fed HFD to induce NAFLD, infiltration of immature macrophages that are CD11b^+^Ly6C^hi^Ly6G^-^ are observed. These macrophages are more readily able to produce proinflammatory cytokines than those from the lean mice controls (75). In mice fed methionine-choline deficient (MCD) diet to induce NASH, induction of macrophage proinflammatory genes is found to associate with more progressive fibrosis (76). Depletion of macrophages using liposomes to deliver clodronate led to reduced expression of proinflammatory genes and attenuated the progression to NASH and fibrosis in mouse models (32, 77). In genetic models where cytokine signals are manipulated, infiltration of macrophages are also found to prolong liver injury (78). Deletion of *Ccl2* (C-C motif chemokine ligands 2), a chemokine that recruits monocytes to the liver, results in reduced liver damage and fibrosis (76). Treatment with a dual antagonist for CCR2 and CCR5, receptors for CCL2 and CCL5, significantly reduced macrophages and protected rats from liver injury in a diet induced NASH model (79) and has shown promising effects for NASH in phase II clinical trial (80). Inhibiting activation of Kupffer cells and infiltration of monocytes by deletion of proinflammatory receptor Trem-1 also significantly attenuated liver inflammation, injury and liver fibrosis induced by CCL4 treatment (81). Adoptive transfer of Trem1-sufficient Kupffer cells led to reactivated inflammation and injury, suggesting that the presence of Trem1-sufficient Kupffer cells can sustain chronic inflammation. In both CCL4 induced liver injury and MCD feeding induced NASH mice, pharmacological inhibition, or deficiency of monocyte chemoattractant protein (MCP-1 or CCL2) led to reduced liver injury and inflammation (76, 82). Together, inflammation is thought to be a crucial phase for the disease progression of NAFLD and the role of macrophages appear to be important in this progression.

### Steatosis induced inflammation establishes a pro-tumor microenvironment

Chronic injury and the associated inflammatory responses are a major link between liver steatosis and cancer development. The development of liver cancer is a slow process that evolves from premalignant lesions developed within chronically damaged livers (83). In chemical induced hepatocarcinogenesis, HFD feeding promotes hepatic inflammation and exacerbates tumor development (84). In HCC mice induced by transgenic expression of hepatitis C virus core protein, HFD feeding to induce liver steatosis significantly increased tumor incidence (85). In these mice, the toll-like receptor (TLR) signal involved in innate immune response was found to promote the transformation of liver tumor initiating cells (86). In mice lacking p53 and concurrent expression of c-Myc, T cell mediated immune surveillance was found to reduce tumor formation and increase survival. This tumor surveillance is overcome when the β-catenin pathway is induced by exogenous expression of active β-catenin, further confirming that β-catenin signal sustains tumor growth (87). In the *Pten* deletion model, steatosis is required for tumor growth and is accompanied by inflammation and induction of β-catenin (33, 88). It was discovered that depletion of macrophages reduces Wnt/β-catenin signals and attenuates tumor growth (32, 89). Together, these studies suggest steatosis establishes an inflammatory environment that is pro-tumor growth.

Infiltration and reprogramming of macrophages are observed in essentially all experimental models and HCC patients. In HFD fed mice where tumors are initiated by DEN treatment, macrophage recruitment accompanied chronic liver injury and liver cancer development (84). In genetic models of NAFLD-NASH-liver cancer, macrophages also play a dominant role in promoting liver cancer development. Depletion of macrophages resulted in reduced tumor incidence in the *Pten* deletion mice (32) and this was thought to involve TLR signaling (90). Together, this evidence suggests that while macrophages can produce pro-repair cytokines, sustained presence of macrophages can prolong liver injury and result in further liver damage.

During liver repair in response to injury, liver macrophages, particularly Kupffer cells are credited in producing pro-mitogenic cytokines to induce the growth of liver progenitor cells and promote liver regeneration (91–93). Depletion of macrophages attenuates tissue repair and resulted in exacerbated fibrogenic phenotype (92) and also led to delayed recovery of metabolic functions performed by the liver (94). When inflammation is not resolved, the signals produced by macrophages exacerbate liver injury and lead to chronic inflammatory conditions and sustain the production of proinflammatory cytokines (6, 65). During liver tumorigenesis, the chronic inflammatory condition and proinflammatory cytokines promote tumorigenesis by providing the tumor microenvironment as well as signaling the growth and promoting the proliferation of tumor initiating cells (91). High fat diet feeding induces macrophage production of a number of inflammatory factors and cytokines including interleukins, C-C ligands (CCLs), interferon γ (IFNγ) and tumor necrosis factor α (TNFα) to facilitate hepatocyte proliferation (84). Cytokines produced by these resident as well as infiltrating macrophages such as TNFα, transforming growth factor β (TGF-β), interleukin 6 (IL-6) and 18 (IL-18) are highly associated with the development and progression of hepatocellular carcinoma (HCC). In a mouse tumor model established by subcutaneous transfer of DEN-initiated liver tumor initiating cells, depletion of macrophages attenuated the progenitor cell properties and reduced tumor development (95). The presence of macrophage-produced TNFα also triggers chromosomal instability in liver tumor initiating cells, permitting propagation of genotoxic events leading to tumorigenesis (95). TNFα produced by macrophages are also proposed to promote the proliferation of liver cancer initiating cells (96). These tumor initiating cells were found to display similar transcriptome profiles as the ov-6 positive liver progenitors that express LIN-28 (97). The expression of LIN-28 allows these cells to respond to the interleukin-6 (IL-6) signal to proliferate. In MCD diet fed mice, macrophage reprograming also contributed to the proliferation of liver progenitors and promoted HCC proliferation (98). In tumors induced by expression of Myc and deletion of TP53, upregulation of β-catenin promoted immune escape of the tumors involving defective recruitment of myeloid lineage cells that include macrophages (87). As a potential driver mutation gene, activation of β-catenin is associated with liver tumor initiation (27, 48). In the *Pten* deleted NAFLD-NASH-Tumor mice, β-catenin was found necessary to sustain the growth of liver tumor initiating cells as deletion of β-catenin attenuated their growth (32, 33, 88). Depletion of macrophages suppressed Wnt/β-catenin signal and led to reduced tumor burden in these mice. TLR4 was found to play a role in the macrophage-promoted proliferation of tumor initiating cells and tumorigenesis in these mice (90). Together, these data suggest that macrophages may be necessary to both sustain tumor initiating cell proliferation as well as establishing the liver injury environment that allows the tumors to grow.

## Cytokines in steatosis driven HCC

**Figure 3:**
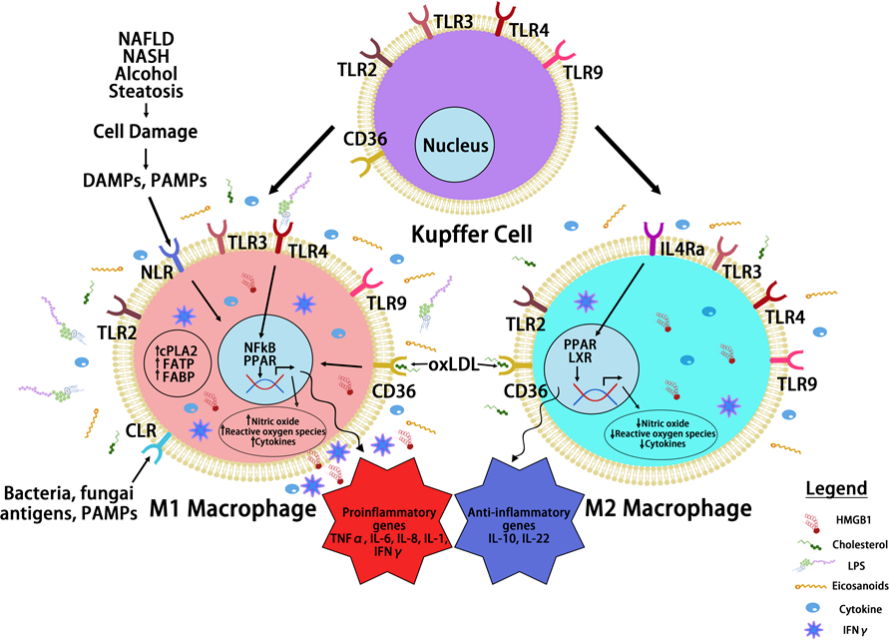
Macrophage Reprogramming in Steatosis Driven HCC. During liver inflammation, Kupffer cells and macrophages express scavenger receptors (SR) and pattern recognition receptors (PRR) to respond to pathogens and liver damages. Activation of PRR receptors by pathogen activated molecular pattern (PAMP) and damage activated molecular pattern (DAMP) molecules reprograms hepatic macrophages to produce inflammatory cytokines/chemokines. The binding of PRRs and SRs to steatotic induced PAMP and DAMPs reprograms hepatocyte macrophages. The reprogramed M1 macrophages produce a proinflammatory cytokines where the reprogrammed M2 macrophages produce anti-inflammatory cytokines to mediate the progression of steatosis to cancer. Toll like receptor (TLRs) and NOD-like receptors (NLRs) are two common PRRs used by PAMP and DAMP to induce macrophage reprogramming. Cluster of differentiation 36 (CD36) belongs to SR family of receptors and binds to oxidized LDL. Other PRR receptors include the C-type lectin receptors (CLRs) is also expressed on the reprogrammed macrophages. Binding of these receptors to their ligands such as lipopolysaccharide (LPS), high mobility group box 1 (HMGB1) and oxidized low density lipoprotein (oxLDL) activates the innate immune response and produce cytokines and chemokines that play important roles in tumorigenesis. It also activates nuclear factor kappa B (NF-κB) and proliferator-activated receptor (PPAR) and other liver nuclear receptors such as liver X receptor (LXR) regulates transcriptional reprograming of these macrophages.

Inflammatory cytokines play key roles in the communication between macrophages with surrounding cell types and also reprograming macrophages to different spectrums of polarizations under given stimulatory conditions, resulting in high heterogeneity of liver macrophages (99). Beyond proliferation of resident Kupffer cells and infiltration of monocyte-derived macrophages, hepatic macrophages are also stimulated or “reprogrammed” to produce a variety of pro- and anti-inflammatory cytokines that classify them on the spectrums of M1 vs. M2 polarization (**Figure 3**). During steatosis driven liver cancer development, a complex interaction of anti- and pro-inflammatory cytokines promotes cell proliferation and activation of HCC progenitor cells and results in cancer promotion (95, 97). Like macrophages themselves, these cytokines play dual roles in liver cancer development by 1) promoting proliferation of cancer cells and 2) exacerbating liver injury to produce a protumor microenvironment.

### Proinflammatory cytokines

Several proinflammatory cytokines appear to be induced during the development and progression from steatosis to liver injury to cancer (71, 75, 90, 100–106). In patients with chronic inflammatory and fibrotic liver diseases, analysis of classical CD14^++^CD16^-^ monocytes in the liver found that they express both macrophage and dendritic cell markers with a high capacity for phagocytosis, antigen presentation, and regulatory T cell proliferation (103). They also secrete proinflammatory cytokines including TNFα, IL-6, IL-8 as well as IL-1 consistent with a role in the wound healing response where proinflammatory cytokines induce hepatocyte proliferation for tissue repair. In mice fed a Western diet, tumor progression is associated with a predominant M1 proinflammatory cytokine vs. the M2 pattern (83). In ALD, severe liver damage is also accompanied by significantly elevated M1 proinflammatory macrophage marker expression in C57Bl/6 mice, whereas less damage is observed in Balb/c mice where no change of M1 markers is found (104). In morbidly obese patients with NAFLD, reduced liver M2 anti-inflammatory macrophage marker expression (increased M1/M2 ratio) is associated with more severe steatosis. This reduced M2 macrophage phenotype also correlated with increased hepatocyte cell death and elevated serum levels of alanine aminotransferase (ALT), a clinical index of liver injury (107). Together, the proinflammatory cytokines secreted by macrophages in steatotic liver establishes a pro-inflammatory tissue microenvironment that can promote further liver damage and sustained inflammation.

One of the proinflammatory cytokines produced with steatosis, TNFα, plays a key role in liver carcinogenesis (16, 108). TNFα is a pleiotropic cytokine produced by many cell types with monocyte lineage cells being the primary source. In the liver, both Kupffer cells and infiltrating monocytes can produce TNFα in response to stimulation. TNFα produced by macrophages was found to promote cancer cell sphere formation *in vitro* (95). In this study, TNFα enhances the self-renewal abilities of the cancer cells. Consistently, in MUP-uPA mice fed with HFD, development of NASH and HCC are dependent on macrophage secreted TNFα. Knocking down TNFα Receptor 1 (TNFR1) significantly reduced liver damage and tumor formation (109). NFκB signal is implicated in this TNFR1 mediated hepatocyte death as deletion of IKKβ or NEMO, two NFκB signal modulators resulted in spontaneous progression of TNFα mediated hepatitis to cancer (110). In a DEN induced tumor model, deletion, or inhibition of TNFα resulted in reduced tumor incidence accompanied by suppressed activation and proliferation of hepatic progenitors *via* the TNFR2-STAT3 pathway (111). Consistent with a role of TNFα in liver regeneration, hepatocyte growth is also inhibited, resulting in a shorter lifespan even though tumor burden was reduced. In CCA, this effect of TNFα signal in chronic liver injury was shown to be mediated by JNK signaling and involves mitochondrial reactive oxygen species (ROS) production (112).

It was determined that hepatic IL-6 expression is significantly increased in the livers of patients with NASH (113). IL-6 signals through two pathways on target cell: classical signaling involves IL-6 binding to its receptor IL-6R on target cells. In the absence of IL-6R, IL-6 trans-signaling is induced, which involves an IL-6 binding to cleaved and soluble IL-6R provided by surrounding cells (114). During hepatocellular carcinogenesis, IL-6 trans-signaling pathway, rather than the IL-6 classic signaling contributes to the development of tumors by enhancing tumor proliferation through STAT3 and β-catenin activation and stimulating endothelial cell proliferation to promote tumor angiogenesis (115). Furthermore, IL-6 induces pre-cancerous progenitor cell proliferation and transformation into tumor initiating cells (97). IL-6 treatment *in vitro* led to early S phase entry in H4IIE HCC cells as shown by the reduced G0/G1 phase after treatment (116). IL-6 also contributes to the drastically different HCC incidence in male vs female mice treated with DEN (117). Recruitment of tumor-associated macrophages by the Yes-associated protein YAP, an oncogene overexpressed in a subset of HCC patients, also involves IL-6 signaling (118). Similar to the role of TNFα, IL-6 signals through STAT3 protect from chronic liver injury. However, the role of IL-6 in liver injury and tumorigenesis is also context dependent as IL-6 also protects from liver injury by promoting hepatocyte regeneration. In the multidrug-resistant gene 2 knockout (Mdr2^-/-^) mice where 50% of the mice develop tumors after chronic injury, IL-6 signal deficiency led to more severe steatosis and inflammation presumably due to the inability of hepatocyte regeneration/increased hepatocyte apoptosis after injury (101). Regardless, the resulting infiltration of macrophages promoted tumor growth and led to increased tumor burden (101, 119).

Other proinflammatory cytokines including IL-1, IL-8, IL-17, IL-18 and IFNγ may also be produced by macrophages to play similar roles in liver regeneration and sustain tumor cell growth. Like IL-6 and TNFα, IL-1 is commonly induced in steatotic livers when macrophage proliferation and infiltration are induced (17, 77, 84, 120) and is necessary for the whole spectrum of pathologies associated with steatosis, injury and cancer (104, 106). The expression of C-X-C receptor 2 (CXCR2), a receptor for IL-8, is upregulated in both HCC and iCCA. Targeting inhibition of CXCR2 results in reduced proliferation in Huh7 and HepG2 cells (121). The induction of liver IL-8 also provides signals for breast cancer cells to escape dormancy when they metastasize to the liver, suggesting that IL-8 indeed establishes a protumor environment in the liver (122). Blockade of IL-17 was shown to protect from liver injury including injuries induced due to steatosis (123). While macrophages may or may not be the primary source of IL-17 (124, 125), IL-17 does induce hepatic macrophage production of IL-6 and TNFα (126, 127). IL-18 is produced by THP-1 macrophages together with IL-1 in cultures exposed to hepatitis C virus (128). In the liver, administration of recombinant IL-18 induces severe liver injury concurrent with induced IFNγ secretion from NK cells (129). Delivery of neutralizing antibody targeting IL-18 reduced serum ALT levels and liver inflammation. Together, the proinflammatory cytokines produced by Kupffer cells and infiltrating monocyte derived macrophages establishes a sustained inflammatory environment to promote the growth of hepatocytes. This proinflammatory environment also acts on tumor initiating cells to propagate the genotoxic events, leading to tumor development.

### Anti-inflammatory cytokines

Macrophage polarization was defined by IL-1β/iNOS producing macrophages as M1 and Arg-1/IL-10 expressing macrophages as M2 phenotypes. As the defining M2 cytokine, IL-10 is one of the best documented anti-inflammatory cytokines. In HFD feeding or alcohol induced liver injury, IL-10 is also induced (84, 108). It was proposed that the Kupffer cell production of IL-10 is also pro-regeneration and pro-survival for the hepatocytes (130). During the initial stage of chronic liver damage, liver macrophages also express C-X-C Ligand16 (CXCL16) to recruit NKT cells (131). This results in the formation of NKT and Kupffer cell clusters during liver steatosis. Clustered NKT-Kupffer cells secrete IFNγ and IL-10 (23). Another IL-10 family of cytokine, IL-22 also plays a role in NASH driven hepatocarcinogenesis. IL-22 levels gradually increase 5 months after the start of DEN treatment. It was concluded that continuous activation of STAT3 and CyclinD1 sustained IL-22 promoted cell proliferation (132). More recently, metformin, the antidiabetic drug was found to promote cell apoptosis through activation of Hippo signaling and to inhibit IL-22 induced tumor cell proliferation and invasion (133).

### Chemokines

Chemokines are released by Kupffer cells, liver sinusoidal endothelial cells and hepatic stellate cells to recruit infiltrating immune cells (134). Chemokine levels and their receptors are elevated in tissue and blood samples from patients with NASH and HCC compared with healthy and non-tumor controls (135–142). Among the two primary groups of chemokines, CC chemokines (CCLs) are known for their ability to recruit monocytes and lymphocytes, while CXC chemokines (CXCLs) are potent neutrophil attractants and can promote angiogenesis (143).

Upon ligand binding, Kupffer cells release CCL2 to recruit monocytes (144). In *Ccl2* deletion mice, reduced inflammatory cell infiltration is observed (76). Inhibition of CCL2 with an RNA oligonucleotide that binds to CCL2 or neutralizing antibody for CCL2 led to reduced monocyte chemotaxis and reduced macrophage infiltration into the liver (82). These treatments resulted in reduced production of TNFα and IL-6, two macrophage produced cytokines. In NAFLD and NASH livers, macrophages also upregulate CCL3 and this induction of CCL3 facilitates macrophage infiltration and production of proinflammatory cytokines (135).

Kupffer cells also release CXCL1,CXCL2 and CXCL8 to recruit neutrophils (144). In HFD+Alcohol induced liver steatohepatitis, blockage of CXCL1 was found to reduce hepatic neutrophil infiltration and significantly inhibit liver injury (145). CXCL2 induction was shown to play a pivotal role in the recruitment of neutrophils in ConA induced hepatitis (146). In cholestatic patients, upregulation of CXCL8 and its receptors CXCR1/2 is associated with neutrophil infiltration whereas macrophage infiltration is associated with CXCL8 signal upregulation in non-cholestatic patients (147). This upregulation of CXCL8 signal plays important roles in the tumor microenvironment (122, 142). Furthermore, the macrophage derived CXCL9 and 10 are required for immune checkpoint therapy to block the infiltration of CD8+ T cells (148). The release of CXCL10 from macrophages is induced by steatosis (149) and deficiency of macrophage lipid receptor CD36 led to reduced release of CXCL10 in the liver (150). The role of CXCL5 in steatosis and liver cancer has drawn attention recently (151). Hepatic CXCL5 expression was higher in patients with severe fibrosis and cirrhosis (141). Multivariate Cox analysis of TCGA data identified that among 110 differentially expressed genes that were associated with HCC overall survival, CXCL5 and IL18RAP were the only 2 genes that predict the prognosis independently (142).

## Macrophage reprogramming in steatosis driven HCC

**Figure 4:**
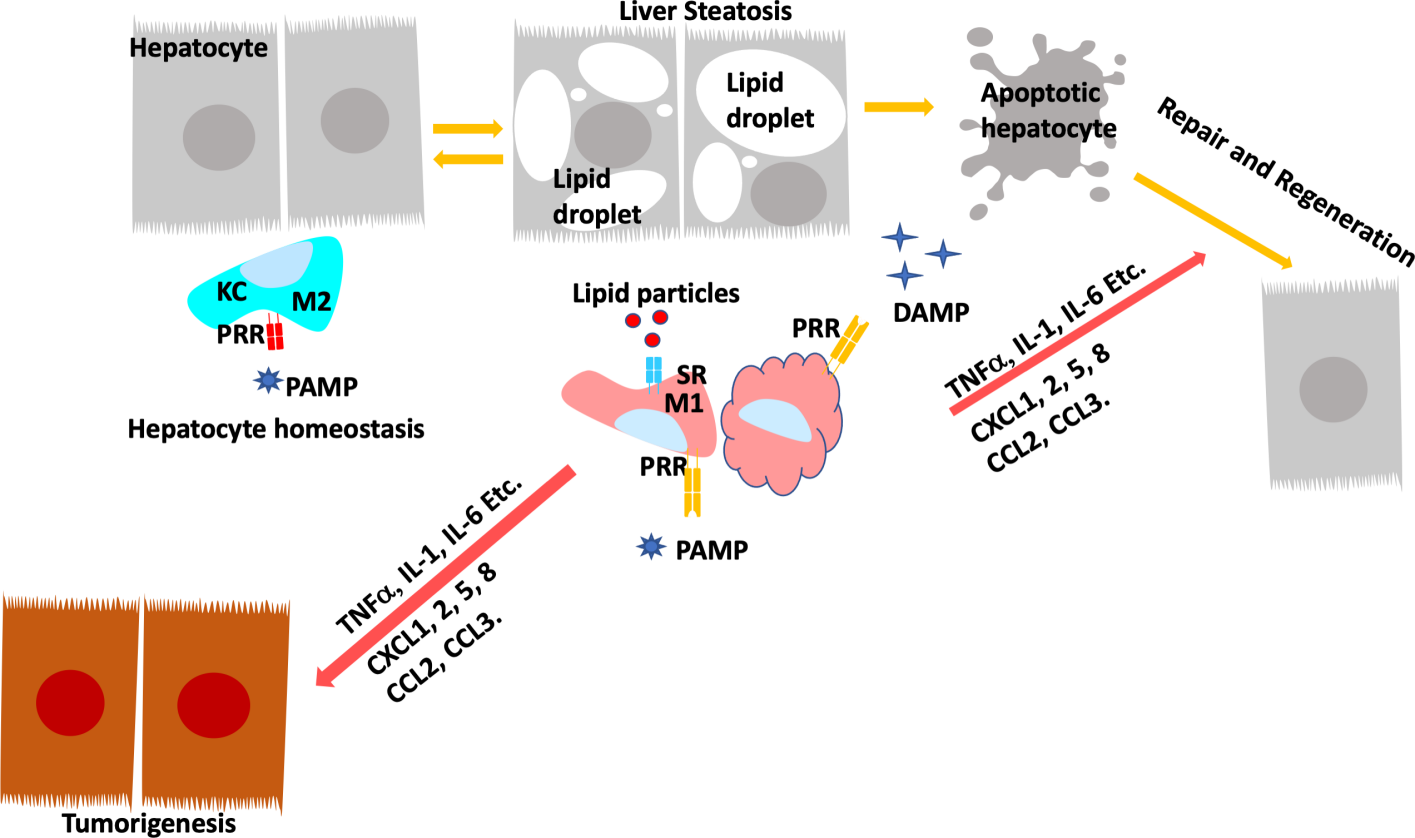
Programing of Hepatic Macrophages by PAMP and DAMP *via* PRR and SR. In normal, Kupffer cells recognize pathogen induced molecular patterns (PAMP) such as LPS coming through the portal vein. Kupffer cells clears these bacterial toxins without inducing inflammation to maintain hepatocyte homeostasis. During steatosis and steatotic injury, PRRs also bind to damage induced molecular patterns (DAMP) released by hepatocytes. The chronic injury induces proinflammatory responses from Kupffer cells as well as infiltrating macrophages. In addition, particles released by steatotic hepatocytes are also taken up by macrophages *via* scavenger receptors (SR). The binding of DAMP and lipid particles to PRR and SR induces the release of proinflammatory cytokines and chemokines including TNFα, IL-1, IL-6, CCL2 and 3 as well as CXCL1,2,5, and 8. These inflammatory mediators signals tissue repair and also promotes genotoxic events in liver cancer.

The crosstalk of macrophage with hepatocytes is crucial for sustaining inflammatory signals during liver injury. In normal livers, macrophages contribute to normal hepatocytes function by regulating glucocorticoid signals (152). During liver inflammation, Kupffer cells and infiltrating macrophages express scavenger (SR) and pattern recognition receptors (PRR) to readily respond to pathogens and liver damage (153). Activation of PRR receptors by pathogen activated molecular pattern (PAMP) and damage activated molecular pattern (DAMP) molecules produced primarily by hepatocytes reprogram hepatic macrophages to produce inflammatory cytokines/chemokines that reverse the immune-suppressive liver environment and facilitate tissue repair (154). Scavenger receptors (SRs) are defined as macrophage receptors for modified lipids in foam cell formation but can also bind to other bioactive ligands (155). While binding of PRRs to ligands induces the release of pro- and anti-inflammatory cytokines and chemokines, uptake of modified lipids *via* SRs also leads to removal of the pathogen/damaged cells that present the recognized molecular patterns in addition to releasing inflammatory mediators (**Figure 4**).

### Pattern recognition receptors

Macrophages possess a number of different receptors that recognize intracellular and extracellular PAMPs and DAMPs as well as membrane bound ligands. This includes the TLR family of membrane receptors that play key roles in both innate and adaptive immune response. A cytosolic nucleotide-binding domain and leucine-rich repeat containing receptors (NOD-like receptors, NLRs) is another super family of PRR that is responsible for inflammasome activation which is essential for a successful immune response. The C-type receptors (CLRs) at the cell membrane recognize foreign antigens including bacterial and fungal antigens. Other PRRs including the 5’-triphosphate-RNA and dsRNA RIG-I-like receptors, as well as several DNA cytosolic sensors are also expressed in the liver microenvironment.

In NAFLD and ALD, steatosis induces chronic injury and hepatocyte damage. The damaged hepatocytes are the major source for DAMPs in the steatotic liver. For example, bile acid accumulation in hepatocytes triggers the assembly of NLR protein 3 inflammasome and the subsequent release of IL-1β that can bind to IL-1 receptors on macrophages. The TLR family members are high-affinity transmembrane receptors expressed on macrophages including Kupffer cells (156). The engagement of TLR4 with LPS triggers the sequential release of proinflammatory cytokines including TNF, IL-1, and IFN-β and other proinflammatory mediators such as the high mobility group box 1 (HMGB1) (157). During fatty liver diseases, free fatty acids also induce HMGB1 overexpression and secretion from hepatocytes. HMGB1 binds and activates TLR4 receptors on Kupffer cells and induce the release of proinflammatory cytokines such as TNFα and IL-6 (158). Similarly, during HFD feeding, hepatocytes release mitochondrial DNAs which stimulate Kupffer cell TLR9 receptors and subsequent TNFα secretion. Cholesterol laden lipid droplets formed within hepatocytes can also activate Kupffer cells through direct contact, this promotes IL-1β secretion in these Kupffer cells (159).

### Scavenger receptors

The distinct characteristic of steatotic liver injury is lipotoxicity. The accumulation of lipids in hepatocytes results in metabolic and oxidative stress that not only results in hepatocyte apoptosis but also directly signals inflammatory responses *via* macrophage cell surface receptors (55, 160, 161). During the pathogenesis of atherosclerosis, plaque formation is induced by the foam cells formed when macrophages scavenge modified low-density lipoproteins (LDL) and deposit them into the endothelial linings of blood vessels. Brown and Goldstein identified SR that are responsible for uptake of the modified LDLs (155). The family of scavenger receptors now are diverse and bind other DAMPs and PAMPs as well. In addition to modified and unmodified LDLs, other lipids such as cholesterols and phospholipids, bacterial pathogens, oxidative particles, and apoptotic cells are all scavenged by macrophages *via* these scavenger receptors (155). In NASH induced by feeding of Western diet, deletion of macrophage scavenger receptor MSR or type B1 scavenger receptor CD-36 led to reduced inflammation likely due to their effects on intracellular cholesterol trafficking in Kupffer cells (162, 163). In LDL receptor deficient (ldl4-/-) mice fed HFD, loss of CD36 or MSR resulted in reduced hepatic inflammation (162). In ConA induced liver injury, it was shown that CD36 sustains inflammation and expression of proinflammatory cytokines and is required for C-X-C ligand 10 induced apoptosis of hepatocytes (150).

Uptake of cholesterol *via* these SRs reprograms liver X receptor (LXR) regulated transcription in macrophages and attenuates the expression of anti-inflammatory genes (164, 165). In addition, the expression of macrophages CD36 and SR-B2 are also subjected to the transcriptional regulation by the orphan nuclear receptor peroxisomal proliferator-activated receptor (PPAR) (166–168). In THP-1 macrophages, it was shown that the downregulation of CD36 in macrophages likely resulted from reduced PPARγ regulated transcription when ratio of n-6/n-3 polyunsaturated fatty acids (PUFAs) is reduced (169). PPARγ has long been recognized as a potential receptor for PUFA produced eicosanoids (170). These effects of PUFAs on CD36 expression and the function of macrophages to produce inflammatory metabolites are at least partially mediated through the activation of PPARs by the bioactive eicosanoids produced from PUFAs (171–173). In fact, cyclooxygenase 2 (COX-2), one of the enzymes metabolizing PUFAs to eicosanoids is only expressed in tissue and infiltrating macrophages in the healthy liver (174). Activation of PPARγ by eicosanoids was found to sustain the production of TNFα, IL-1 and IL-6 induced by LPS and induce IL-10 downregulation in macrophages (175). In HFD induced NAFLD, loss of CD47, an inhibitor for macrophage activation and phagocytosis, leads to increased production of proinflammatory cytokines involving activation of PPARα (176). In Kupffer cells, LPS treatment induced TNFα and IL-6 is attenuated by PPAR agonist rosiglitazone (177). Thus, *via* regulation of PPARs, macrophages scavenge lipid particles to produce both pro- and anti-inflammatory cytokines (160). These PUFA derivatives including prostanoids, leukotrienes, HETES, EETs and lipoxins have all been indicated to promote a protumor inflammatory environment (178). During hepatocarcinogenesis, inhibiting COX-2 and epoxide hydrolase led to reduced “cytokine and eicosanoid storm”, resulting in cancer prevention (179). The treatment with lipoxin A4, a pro-resolving eicosanoid in inflammation, led to reduced HCC proliferation induced by activated macrophages (180). Together, macrophage engulfment of lipids *via* the scavenger receptors will result in increased production of PUFA derived eicosanoids. These eicosanoids can be inflammatory mediators on their own and induce the production of inflammatory cytokines/chemokines *via* the transcriptional activities of nuclear receptors such as PPAR and others. By producing the proinflammatory eicosanoids and cytokines, macrophages/Kupffer cells establish a pro-tumor microenvironment in the injured livers of NAFLD and NASH (181–183).

## The therapeutic potential of targeting steatosis for liver cancer treatment

Pathologically, 80% of liver cancer occurs in patients with underlying liver disease that displays lipid metabolic dysfunctions known as liver steatosis (184), a condition that develops in all obese individuals and is commonly associated with liver cancer (5). In a zebra fish model of HCC promoted by HFD, metformin the first line drug used for treatment in diabetes, reduced TNFα expressing pro-inflammatory macrophages leading to increase T-cell population in the livers, and inhibited cancer progression (185). In mouse HCC induced by DEN treatment, metformin treatment reduced the number of foci. This reduction was thought to be an effect of lowered hepatic expression of interleukin-22 and inhibition of YAP phosphorylation (133). The binding of metformin directly to the C-terminal of HMGB1 may also play roles in its anti-inflammatory and tumor suppressive functions (186). Statin, a cholesterol lowering drug has been proposed as treatment for chronic liver disease (187). NAFLD patients who take more than 600 cumulative daily doses of statin had a 70% reduction in hazards of developing HCC (HR, 0.30; 0.20-0.43) (188). Longer usage of more than 5 years and higher doses reduced the rate of NASH related HCC by 24-35% (189, 190). Both Metformin and Statin may target AMPK for their lipid reduction function (191). In a HFD model treated with DEN, AMPK activator reduced tumorigenesis and IL-6 signaling in the liver (192). Activation of AMPK also suppresses HCC progression and metastasis induced due to deficiency of FATP5 (fatty acid transporter protein 5) (193). Loss of the upstream kinase, LKB1 that phosphorylates and activates AMPK was also found to synergize with *Pten* loss to promote liver cancer development (194). Indeed, Sorafenib, the first line targeted therapy for HCC suppresses NASH through mechanisms involving alteration of mitochondrial uncoupling and subsequent activation of AMPK (195). These observations indicated that mitochondrial metabolism is an underexplored mechanism that may provide potential targets for HCC treatment as LKB-AMPK acts as primary cellular sensors of energy crisis to promote ATP production. Consistently, plasmas from NASH patients were found to contain high levels of mitochondrial DNA and these mitochondrial DNA signal through TLR9 to regulate hepatic inflammation, acting as a potential mechanism for how steatosis establishes the proinflammatory tumor microenvironment. In addition, targeting mitochondrial functions attenuates steatosis and inflammation in the liver (196, 197). Together, this evidence suggests that targeting steatosis *via* reducing lipid burden and/or altering mitochondrial function can impact liver cancer development.

The majority of liver cancer patients are diagnosed in the advanced stages of the disease, eliminating surgery or transplantation the only curative treatment for liver cancer. In patients with advanced disease, the combination of immune checkpoint (CPI) therapy such as anti PD-L1 antibody atezolizumab and the VEGF antibody bevacizumab has become the new standard of care. PD-L1 is highly expressed by liver macrophages in the tumor stroma (198). These macrophages repress the tumor-specific CD8 T-cell activity and induce their apoptosis through the Fas receptors to promote tumor growth (199, 200). Furthermore, Kupffer cells also stimulate the proliferation of antigen specific CD4^+^ Tregs and their release of IL-10 to inhibit the activities of cytotoxic T lymphocyte (91, 92). Additionally, prostaglandins produced by Kupffer cells may inhibit T cell activation (201–203). Together, activation of hepatic macrophages and their expression of PD-L1 appears to promote tumor escape by inducing an immune tolerance and reduce immune surveillance.

Patients with NASH and ASH respond poorly (median survival 5.4 months) to CPIs compared to those without steatosis (Median survival 11 months) (204). Given that CPI blocks the ability of macrophage/Kupffer cells to induce immunosuppressive environment in the liver, identifying the hepatic macrophage produced factors that allow the liver to escape this immune surveillance may be a key to future therapeutic development targeted at inflammatory tumor microenvironment associated with steatosis. As DAMP and PAMP that are present in the NAFLD/NASH livers, PRR and SR signals that controls the macrophage response to DAMP and PAMP are considered potential targets of intervention. A promising dietary intervention is n-3 fatty acids. Treatment with n-3 fatty acids was shown to inhibit both protein and mRNA levels of CD36 whereas n-6 fatty acids activate both (205, 206). In fat-1 transgenic mice fed STZ/HFD to induce NASH, ubiquitous expression of n-3 desaturase converts n-6 PUFAs to n-3 PUFAs and led to downregulation of CD36 and reduced liver damage (207). These dietary intervention studies suggest that targeting PRR and SR may be promising to reduce the tumor microenvironment and may work together with CPI to attenuate tumor growth in the liver.

One interesting discovery in CPI resistance is the role of the Wnt/β-catenin signal. The Wnt/β-catenin signaling pathway plays versatile roles in liver metabolism and tumorigenesis (32, 48, 208) due to its varied functions in different cell types in the liver. As such, upregulation of β-catenin allows tumors to escape CPI therapy and is one of the signals highly associated with CPI resistance together with steatosis (87). Interestingly, steatosis was found to induce macrophage expression of Wnt and the Wnt/β-catenin signaling mediates tumorigenesis in mouse models (32, 88). Thus, the induction of Wnt in macrophages by steatosis may play a role in the immune escape of these tumors. Further studies to elucidate how steatosis induces Wnt upregulation in macrophages is necessary to understand the resistance of steatosis associated liver cancer to CPI treatment.

Overall, liver cancer is the 6th most common type of cancer and the second leading cause of cancer deaths in the world with a median 10-year survival of just 11 months (209–211). In the liver, cancer development is highly associated with the development of steatosis and inflammation. Innate immune system and particularly Kupffer cells, the residence macrophages, act as the first responders following steatotic liver injuries. As such, targeting steatosis that show promising results in attenuating liver inflammation holds great potential in further therapeutic development as treatment of liver cancer (**Table 1**). Additionally, steatosis hinders CPI responses partially due to their effects on macrophages and macrophage production of inflammatory signals. Understanding how Kupffer cell are reprogramed to interact with innate immune system during the progression of steatosis is crucial for future therapeutic development targeted at overcoming resistance to current liver cancer therapy. Finally, identifying signals within tumor cells that respond to these protumor inflammatory signals produced by macrophages will result in novel therapeutic target that can overcome resistance to immunotherapy. In summary, targeting macrophages and macrophage interaction with tumor cells will provide therapeutic potential for steatosis-driven liver cancer treatment.

**Table 1:** Current Therapy for HCC Treatment and Effects of Potential Lipid Modifying Therapy.

Current Treatment	Advantage	Disadvantage
Resection	Potential currative	Many patients are diagnosed late
Sorafenib	Targeted	Poor response rate
CPI	Advanced patients	Steatosis interfers with response
Potential lipid metabolic targeting AMPK and mitochondrial function		
Metformin	Promising mouse studies	
Statin (600 daily dose)	70% reduction in hazards of developing HCC	

## Author contributions

TT organized the writing of this manuscript. BLS edited the final content of the manuscript. All other authors contributed to either the writing or the art work of this manuscript and are listed alphabetically.

## Funding

BLS acknowledged funding from NIDDK DK131492 and NILM RML013315.

## Conflict of interest

The authors declare that the research was conducted in the absence of any commercial or financial relationships that could be construed as a potential conflict of interest.

## Publisher’s note

All claims expressed in this article are solely those of the authors and do not necessarily represent those of their affiliated organizations, or those of the publisher, the editors and the reviewers. Any product that may be evaluated in this article, or claim that may be made by its manufacturer, is not guaranteed or endorsed by the publisher.

## References

[1] Calle EE Rodriguez C Walker-Thurmond K Thun MJ. Overweight, obesity, and mortality from cancer in a prospectively studied cohort of U.S. adults. New Engl J Med (2003) 348(17):1625–38. doi: 10.1056/NEJMoa021423 12711737

[2] Schutte K Bornschein J Malfertheiner P. Hepatocellular carcinoma–epidemiological trends and risk factors. Digestive Dis (Basel Switzerland) (2009) 27(2):80–92. doi: 10.1159/000218339 19546545

[3] Albanes D. Caloric intake, body weight, and cancer: A review. Nutr Cancer (1987) 9(4):199–217. doi: 10.1080/01635588709513929 3299283

[4] Bray GA. Overweight is risking fate. definition, classification, prevalence, and risks. Ann New York Acad Sci (1987) 499:14–28. doi: 10.1111/j.1749-6632.1987.tb36194.x 3300479

[5] Brar G Tsukamoto H. Alcoholic and non-alcoholic steatohepatitis: Global perspective and emerging science. J Gastroenterol (2019) 54(3):218–25. doi: 10.1007/s00535-018-01542-w PMC6394716 30643981

[6] Loomba R Friedman SL Shulman GI. Mechanisms and disease consequences of nonalcoholic fatty liver disease. Cell (2021) 184(10):2537–64. doi: 10.1016/j.cell.2021.04.015 PMC12168897 33989548

[7] Nassir F Rector RS Hammoud GM Ibdah JA. Pathogenesis and prevention of hepatic steatosis. Gastroenterol Hepatol (N Y) (2015) 11(3):167–75. doi: 10.3109/00365521.2015.1030687 PMC4836586 27099587

[8] Tannapfel A Denk H Dienes H-P Langner C Schirmacher P Trauner M. Histopathological diagnosis of non-alcoholic and alcoholic fatty liver disease. Virchows Arch (2011) 458(5):511–23. doi: 10.1007/s00428-011-1066-1 21442288

[9] Jou J Choi SS Diehl AM. Mechanisms of disease progression in nonalcoholic fatty liver disease. Semin Liver Dis (2008) 28(4):370–9. doi: 10.1055/s-0028-1091981 18956293

[10] Schattenberg JM Schuppan D. Nonalcoholic steatohepatitis: the therapeutic challenge of a global epidemic. Curr Opin Lipidol (2011) 22(6):479–88. doi: 10.1097/MOL.0b013e32834c7cfc 22002020

[11] Jeon S Carr R. Alcohol effects on hepatic lipid metabolism. J Lipid Res (2020) 61(4):470–79. doi: 10.1194/jlr.R119000547 PMC7112138 32029510

[12] Lackner C Tiniakos D. Fibrosis and alcohol-related liver disease. J Hepatol (2019) 70(2):294–304. doi: 10.1016/j.jhep.2018.12.003 30658730

[13] Paternostro R Sieghart W Trauner M Pinter M. Cancer and hepatic steatosis. ESMO Open (2021) 6(4):100185–204. doi: 10.1016/j.esmoop.2021.100185 PMC8219773 34139486

[14] Myers S Neyroud-Caspar I Spahr L Gkouvatsos K Fournier E Giostra E. NAFLD and MAFLD as emerging causes of HCC: A populational study. JHEP Rep (2021) 3(2):100231. doi: 10.1016/j.jhepr.2021.100231 33748726 PMC7957147

[15] Kudo A Tanaka S Ban D Matsumura S Irie T Ochiai T. Alcohol consumption and recurrence of non-b or non-c hepatocellular carcinoma after hepatectomy: A propensity score analysis. J Gastroenterol (2014) 49(9):1352–61. doi: 10.1007/s00535-013-0899-6 24136219

[16] Wang Y Ausman LM Greenberg AS Russell RM Wang XD. Nonalcoholic steatohepatitis induced by a high-fat diet promotes diethylnitrosamine-initiated early hepatocarcinogenesis in rats. Int J Cancer (2009) 124(3):540–6. doi: 10.1002/ijc.23995 PMC2671079 19004024

[17] Ambade A Satishchandran A Gyongyosi B Lowe P Szabo G. Adult mouse model of early hepatocellular carcinoma promoted by alcoholic liver disease. World J Gastroenterol (2016) 22(16):4091–108. doi: 10.3748/wjg.v22.i16.4091 PMC4837428 27122661

[18] Kumamoto R Uto H Oda K Ibusuki R Tanoue S Arima S. Dietary fructose enhances the incidence of precancerous hepatocytes induced by administration of diethylnitrosamine in rat. Eur J Med Res (2013) 18:54. doi: 10.1186/2047-783X-18-54 24321741 PMC4029300

[19] Ribas V de la Rosa LC Robles D Nunez S Segales P Insausti-Urkia N. Dietary and genetic cholesterol loading rather than steatosis promotes liver tumorigenesis and NASH-driven HCC. Cancers (Basel) (2021) 13(16):4091. doi: 10.3390/cancers13164091 34439245 PMC8393403

[20] Duan XY Pan Q Yan SY Ding WJ Fan JG Qiao L. High-saturate-fat diet delays initiation of diethylnitrosamine-induced hepatocellular carcinoma. BMC Gastroenterol (2014) 14:195. doi: 10.1186/s12876-014-0195-9 25410681 PMC4240894

[21] Ramesh G Das UN. Effect of dietary fat on diethylnitrosamine induced hepatocarcinogenesis in wistar rats. Cancer Lett (1995) 95(1-2):237–45. doi: 10.1016/0304-3835(95)03896-5 7656238

[22] Schulze K Imbeaud S Letouze E Alexandrov LB Calderaro J Rebouissou S. Exome sequencing of hepatocellular carcinomas identifies new mutational signatures and potential therapeutic targets. Nat Genet (2015) 47(5):505–11. doi: 10.1038/ng.3252 PMC4587544 25822088

[23] Pinyol R Torrecilla S Wang H Montironi C Pique-Gili M Torres-Martin M. Molecular characterisation of hepatocellular carcinoma in patients with non-alcoholic steatohepatitis. J Hepatol (2021) 75(4):865–78. doi: 10.1016/j.jhep.2021.04.049 PMC12164395 33992698

[24] Ki Kim S Ueda Y Hatano E Kakiuchi N Takeda H Goto T. TERT promoter mutations and chromosome 8p loss are characteristic of nonalcoholic fatty liver disease-related hepatocellular carcinoma. Int J Cancer (2016) 139(11):2512–8. doi: 10.1002/ijc.30379 27511114

[25] Alves-Paiva RM Kajigaya S Feng X Chen J Desierto M Wong S. Telomerase enzyme deficiency promotes metabolic dysfunction in murine hepatocytes upon dietary stress. Liver Int (2018) 38(1):144–54. doi: 10.1111/liv.13529 PMC5741503 28741793

[26] Nejak-Bowen KN Thompson MD Singh S Bowen WC Jr. Dar MJ Khillan J. Accelerated liver regeneration and hepatocarcinogenesis in mice overexpressing serine-45 mutant beta-catenin. Hepatol (Baltimore Md. (2010) 51(5):1603–13. doi: 10.1002/hep.23538 PMC2908905 20432254

[27] Qiao Y Xu M Tao J Che L Cigliano A Monga SP. Oncogenic potential of n-terminal deletion and S45Y mutant beta-catenin in promoting hepatocellular carcinoma development in mice. BMC Cancer (2018) 18(1):1093. doi: 10.1186/s12885-018-4870-z 30419856 PMC6233269

[28] Stauffer JK Scarzello AJ Andersen JB De Kluyver RL Back TC Weiss JM. Coactivation of AKT and beta-catenin in mice rapidly induces formation of lipogenic liver tumors. Cancer Res (2011) 71(7):2718–27. doi: 10.1158/0008-5472.CAN-10-2705 PMC3074499 21324921

[29] Thompson MD Wickline ED Bowen WB Lu A Singh S Misse A. Spontaneous repopulation of beta-catenin null livers with beta-catenin-positive hepatocytes after chronic murine liver injury. Hepatol (Baltimore Md.) (2011) 54(4):1333–43. doi: 10.1002/hep.24506 PMC3184210 21721031

[30] Harada N Miyoshi H Murai N Oshima H Tamai Y Oshima M. Lack of tumorigenesis in the mouse liver after adenovirus-mediated expression of a dominant stable mutant of beta-catenin. Cancer Res (2002) 62(7):1971–7. 11929813

[31] Harvey M McArthur MJ Montgomery CA Jr. Butel JS Bradley A Donehower LA. Spontaneous and carcinogen-induced tumorigenesis in p53-deficient mice. Nat Genet (1993) 5(3):225–9. doi: 10.1038/ng1193-225 8275085

[32] Debebe A Medina V Chen CY Mahajan IM Jia C Fu D. Wnt/beta-catenin activation and macrophage induction during liver cancer development following steatosis. Oncogene (2017) 36(43):6020–9. doi: 10.1038/onc.2017.207 PMC5666317 28671671

[33] Galicia VA He L Dang H Kanel G Vendryes C French BA. Expansion of hepatic tumor progenitor cells in pten-null mice requires liver injury and is reversed by loss of AKT2. Gastroenterology (2010) 139(6):2170–82. doi: 10.1053/j.gastro.2010.09.002 PMC2997180 20837017

[34] He L Gubbins J Peng Z Medina V Fei F Asahina K. Activation of hepatic stellate cell in pten null liver injury model. Fibrogenesis Tissue Repair (2016) 9:8. doi: 10.1186/s13069-016-0045-1 27307790 PMC4908727

[35] Stiles BL Kuralwalla-Martinez C Guo W Gregorian C Wang Y Tian J. Selective deletion of pten in pancreatic beta cells leads to increased islet mass and resistance to STZ-induced diabetes. Mol Cell Biol (2006) 26(7):2772–81. doi: 10.1128/MCB.26.7.2772-2781.2006 PMC1430339 16537919

[36] Aggarwal R Peng Z Zeng N Silva J He L Chen J. Chronic exposure to palmitic acid down-regulates AKT in beta-cells through activation of mTOR. Am J Pathol (2022) 192(1):130–45. doi: 10.1016/j.ajpath.2021.09.008 PMC8759041 34619135

[37] He L Hou X Kanel G Zeng N Galicia V Wang Y. The critical role of AKT2 in hepatic steatosis induced by PTEN loss. Am J Pathol (2010) 176(5):2302–8. doi: 10.2353/ajpath.2010.090931 PMC2861095 20348245

[38] He L Li Y Zeng N Stiles BL. Regulation of basal expression of hepatic PEPCK and G6Pase by AKT2. Biochem J (2020) 477(5):1021–31. doi: 10.1042/BCJ20190570 PMC7437399 32096546

[39] Li Y He L Zeng N Sahu D Cadenas E Shearn C. Phosphatase and tensin homolog deleted on chromosome 10 (PTEN) signaling regulates mitochondrial biogenesis and respiration *via* estrogen-related receptor alpha (ERRalpha). J Biol Chem (2013) 288(35):25007–24. doi: 10.1074/jbc.M113.450353 PMC3757167 23836899

[40] Peng Z Aggarwal R Zeng N He L Stiles EX Debebe A. AKT1 regulates endoplasmic reticulum stress and mediates the adaptive response of pancreatic beta cells. Mol Cell Biol (2020) 40(11):e00031–20. doi: 10.1128/MCB.00031-20 32179553 PMC7225563

[41] Moon BC Hernandez-Ono A Stiles B Wu H Ginsberg HN. Apolipoprotein b secretion is regulated by hepatic triglyceride, and not insulin, in a model of increased hepatic insulin signaling. Arterioscler Thromb Vasc Biol (2012) 32(2):236–46. doi: 10.1161/ATVBAHA.111.241356 PMC3870321 22155452

[42] Stiles B Gilman V Khanzenzon N Lesche R Li A Qiao R. Essential role of AKT-1/protein kinase b alpha in PTEN-controlled tumorigenesis. Mol Cell Biol (2002) 22(11):3842–51. doi: 10.1128/MCB.22.11.3842-3851.2002 PMC133830 11997518

[43] Palian BM Rohira AD Johnson SA He L Zheng N Dubeau L. Maf1 is a novel target of PTEN and PI3K signaling that negatively regulates oncogenesis and lipid metabolism. PloS Genet (2014) 10(12):e1004789. doi: 10.1371/journal.pgen.1004789 25502566 PMC4263377

[44] Zeng N Yang KT Bayan JA He L Aggarwal R Stiles JW. PTEN controls beta-cell regeneration in aged mice by regulating cell cycle inhibitor p16ink4a. Aging Cell (2013) 12(6):1000–11. doi: 10.1111/acel.12132 PMC3838454 23826727

[45] Tu T Chen J Chen L Stiles BL. Dual-specific protein and lipid phosphatase PTEN and its biological functions. Cold Spring Harb Perspect Med (2020) 10(1):a036301. doi: 10.1101/cshperspect.a036301 31548229 PMC6938656

[46] Shearn CT Mercer KE Orlicky DJ Hennings L Smathers-McCullough RL Stiles BL. Short term feeding of a high fat diet exerts an additive effect on hepatocellular damage and steatosis in liver-specific PTEN knockout mice. PloS One (2014) 9(5):e96553. doi: 10.1371/journal.pone.0096553 24818992 PMC4018288

[47] Fan B Malato Y Calvisi DF Naqvi S Razumilava N Ribback S. Cholangiocarcinomas can originate from hepatocytes in mice. J Clin Invest (2012) 122(8):2911–5. doi: 10.1172/JCI63212 PMC3408746 22797301

[48] Liu JJ Li Y Chen WS Liang Y Wang G Zong M. Shp2 deletion in hepatocytes suppresses hepatocarcinogenesis driven by oncogenic beta-catenin, PIK3CA and MET. J Hepatol (2018) 69(1):79–88. doi: 10.1016/j.jhep.2018.02.014 PMC6008184 29505847

[49] Wang J Dong M Xu Z Song X Zhang S Qiao Y. Notch2 controls hepatocyte-derived cholangiocarcinoma formation in mice. Oncogene (2018) 37(24):3229–42. doi: 10.1038/s41388-018-0188-1 PMC6002343 29545603

[50] Chronowski C Akhanov V Chan D Catic A Finegold M Sahin E. Fructose causes liver damage, polyploidy, and dysplasia in the setting of short telomeres and p53 loss. Metabolites (2021) 11(6):394. doi: 10.3390/metabo11060394 34204343 PMC8234056

[51] Farazi PA Glickman J Horner J Depinho RA. Cooperative interactions of p53 mutation, telomere dysfunction, and chronic liver damage in hepatocellular carcinoma progression. Cancer Res (2006) 66(9):4766–73. doi: 10.1158/0008-5472.CAN-05-4608 16651430

[52] Farazi PA Glickman J Jiang S Yu A Rudolph KL DePinho RA. Differential impact of telomere dysfunction on initiation and progression of hepatocellular carcinoma. Cancer Res (2003) 63(16):5021–7. 12941829

[53] Naudin CR Maner-Smith K Owens JA Wynn GM Robinson BS Matthews JD. Lactococcus lactis subspecies cremoris elicits protection against metabolic changes induced by a Western-style diet. Gastroenterology (2020) 159(2):639–51.e5. doi: 10.1053/j.gastro.2020.03.010 32169430

[54] Zhang X Coker OO Chu ESH Fu K Lau HCH Wang Y-X. Dietary cholesterol drives fatty liver-associated liver cancer by modulating gut microbiota and metabolites. Gut (2021) 70(4):761–74. doi: 10.1136/gutjnl-2019-319664 PMC7948195 32694178

[55] Zeng N Li Y He L Xu X Galicia V Deng C. Adaptive basal phosphorylation of eIF2alpha is responsible for resistance to cellular stress-induced cell death in pten-null hepatocytes. Mol Cancer Res (2011) 9(12):1708–17. doi: 10.1158/1541-7786.MCR-11-0299 PMC4351767 22009178

[56] Derdak Z Villegas KA Harb R Wu AM Sousa A Wands JR. Inhibition of p53 attenuates steatosis and liver injury in a mouse model of non-alcoholic fatty liver disease. J Hepatol (2013) 58(4):785–91. doi: 10.1016/j.jhep.2012.11.042 PMC3612370 23211317

[57] Yeh TH Krauland L Singh V Zou B Devaraj P Stolz DB. Liver-specific beta-catenin knockout mice have bile canalicular abnormalities, bile secretory defect, and intrahepatic cholestasis. Hepatol (Baltimore Md. (2010) 52(4):1410–9. doi: 10.1002/hep.23801 PMC2947599 20722001

[58] Zhang XF Tan X Zeng G Misse A Singh S Kim Y. Conditional beta-catenin loss in mice promotes chemical hepatocarcinogenesis: role of oxidative stress and platelet-derived growth factor receptor alpha/phosphoinositide 3-kinase signaling. Hepatol (Baltimore Md. (2010) 52(3):954–65. doi: 10.1002/hep.23747 PMC3100799 20583210

[59] Tan X Yuan Y Zeng G Apte U Thompson MD Cieply B. Beta-catenin deletion in hepatoblasts disrupts hepatic morphogenesis and survival during mouse development. Hepatol (Baltimore Md. (2008) 47(5):1667–79. doi: 10.1002/hep.22225 PMC4449338 18393386

[60] Cabrae R Dubuquoy C Cauzac M Morzyglod L Guilmeau S Noblet B. Insulin activates hepatic wnt/beta-catenin signaling through stearoyl-CoA desaturase 1 and porcupine. Sci Rep (2020) 10(1):5186. doi: 10.1038/s41598-020-61869-4 32198362 PMC7083857

[61] Lai KKY Kweon SM Chi F Hwang E Kabe Y Higashiyama R. Stearoyl-CoA desaturase promotes liver fibrosis and tumor development in mice *via* a wnt positive-signaling loop by stabilization of low-density lipoprotein-Receptor-Related proteins 5 and 6. Gastroenterology (2017) 152(6):1477–91. doi: 10.1053/j.gastro.2017.01.021 PMC5406249 28143772

[62] Tian Y Mok MT Yang P Cheng AS. Epigenetic activation of wnt/beta-catenin signaling in NAFLD-associated hepatocarcinogenesis. Cancers (Basel) (2016) 8(8):76. doi: 10.3390/cancers8080076 PMC4999785 27556491

[63] Fujise T Iwakiri R Kakimoto T Shiraishi R Sakata Y Wu B. Long-term feeding of various fat diets modulates azoxymethane-induced colon carcinogenesis through wnt/beta-catenin signaling in rats. Am J Physiol Gastrointest Liver Physiol (2007) 292(4):G1150–6. doi: 10.1152/ajpgi.00269.2006 17194898

[64] Huang H Lu Y Zhou T Gu G Xia Q. Innate immune cells in immune tolerance after liver transplantation. Front Immunol (2018) 9:2401. doi: 10.3389/fimmu.2018.02401 30473690 PMC6237933

[65] Gao B Seki E Brenner DA Friedman S Cohen JI Nagy L. Innate immunity in alcoholic liver disease. Am J Physiol Gastrointest Liver Physiol (2011) 300(4):G516–25. doi: 10.1152/ajpgi.00537.2010 PMC3774265 21252049

[66] Yona S Kim KW Wolf Y Mildner A Varol D Breker M. Fate mapping reveals origins and dynamics of monocytes and tissue macrophages under homeostasis. Immunity (2013) 38(1):79–91. doi: 10.1016/j.immuni.2012.12.001 PMC3908543 23273845

[67] David BA Rezende RM Antunes MM Santos MM Freitas Lopes MA Diniz AB. Combination of mass cytometry and imaging analysis reveals origin, location, and functional repopulation of liver myeloid cells in mice. Gastroenterology (2016) 151(6):1176–91. doi: 10.1053/j.gastro.2016.08.024 27569723

[68] Heymann F Peusquens J Ludwig-Portugall I Kohlhepp M Ergen C Niemietz P. Liver inflammation abrogates immunological tolerance induced by kupffer cells. Hepatol (Baltimore Md. (2015) 62(1):279–91. doi: 10.1002/hep.27793 25810240

[69] Hwang S Yun H Moon S Cho YE Gao B. Role of neutrophils in the pathogenesis of nonalcoholic steatohepatitis. Front Endocrinol (Lausanne) (2021) 12:751802. doi: 10.3389/fendo.2021.751802 34707573 PMC8542869

[70] Li H Zhou Y Wang H Zhang M Qiu P Zhang M. Crosstalk between liver macrophages and surrounding cells in nonalcoholic steatohepatitis. Front Immunol (2020) 11:1169. doi: 10.3389/fimmu.2020.01169 32670278 PMC7326822

[71] Orfila C Lepert JC Alric L Carrera G Beraud M Vinel JP. Expression of TNF-alpha and immunohistochemical distribution of hepatic macrophage surface markers in carbon tetrachloride-induced chronic liver injury in rats. Histochem J (1999) 31(10):677–85. doi: 10.1023/A:1003851821487 10576417

[72] Jemail L Miyao M Kotani H Kawai C Minami H Abiru H. Pivotal roles of kupffer cells in the progression and regression of DDC-induced chronic cholangiopathy. Sci Rep (2018) 8(1):6415. doi: 10.1038/s41598-018-24825-x 29686325 PMC5913224

[73] Remmerie A Martens L Thone T Castoldi A Seurinck R Pavie B. Osteopontin expression identifies a subset of recruited macrophages distinct from kupffer cells in the fatty liver. Immunity (2020) 53(3):641–57.e14. doi: 10.1016/j.immuni.2020.08.004 PMC7501731 32888418

[74] Friedman SL. Mechanisms of hepatic fibrogenesis. Gastroenterology (2008) 134(6):1655–69. doi: 10.1053/j.gastro.2008.03.003 PMC2888539 18471545

[75] Deng ZB Liu Y Liu C Xiang X Wang J Cheng Z. Immature myeloid cells induced by a high-fat diet contribute to liver inflammation. Hepatol (Baltimore Md. (2009) 50(5):1412–20. doi: 10.1002/hep.23148 PMC2852608 19708080

[76] Galastri S Zamara E Milani S Novo E Provenzano A Delogu W. Lack of CC chemokine ligand 2 differentially affects inflammation and fibrosis according to the genetic background in a murine model of steatohepatitis. Clin Sci (Lond) (2012) 123(7):459–71. doi: 10.1042/CS20110515 PMC3369401 22545719

[77] Stienstra R Saudale F Duval C Keshtkar S Groener JE van Rooijen N. Kupffer cells promote hepatic steatosis *via* interleukin-1beta-dependent suppression of peroxisome proliferator-activated receptor alpha activity. Hepatol (Baltimore Md. (2010) 51(2):511–22. doi: 10.1002/hep.23337 20054868

[78] Karlmark KR Zimmermann HW Roderburg C Gassler N Wasmuth HE Luedde T. The fractalkine receptor CX(3)CR1 protects against liver fibrosis by controlling differentiation and survival of infiltrating hepatic monocytes. Hepatol (Baltimore Md. (2010) 52(5):1769–82. doi: 10.1002/hep.23894 21038415

[79] Lefebvre E Moyle G Reshef R Richman LP Thompson M Hong F. Antifibrotic effects of the dual CCR2/CCR5 antagonist cenicriviroc in animal models of liver and kidney fibrosis. PloS One (2016) 11(6):e0158156. doi: 10.1371/journal.pone.0158156 27347680 PMC4922569

[80] Friedman SL Ratziu V Harrison SA Abdelmalek MF Aithal GP Caballeria J. A randomized, placebo-controlled trial of cenicriviroc for treatment of nonalcoholic steatohepatitis with fibrosis. Hepatol (Baltimore Md. (2018) 67(5):1754–67. doi: 10.1002/hep.29477 PMC5947654 28833331

[81] Nguyen-Lefebvre AT Ajith A Portik-Dobos V Horuzsko DD Arbab AS Dzutsev A. The innate immune receptor TREM-1 promotes liver injury and fibrosis. J Clin Invest (2018) 128(11):4870–83. doi: 10.1172/JCI98156 PMC6205377 30137027

[82] Baeck C Wehr A Karlmark KR Heymann F Vucur M Gassler N. Pharmacological inhibition of the chemokine CCL2 (MCP-1) diminishes liver macrophage infiltration and steatohepatitis in chronic hepatic injury. Gut (2012) 61(3):416–26. doi: 10.1136/gutjnl-2011-300304 21813474

[83] Mirshahi F Aqbi HF Isbell M Manjili SH Guo C Saneshaw M. Distinct hepatic immunological patterns are associated with the progression or inhibition of hepatocellular carcinoma. Cell Rep (2022) 38(9):110454. doi: 10.1016/j.celrep.2022.110454 35235789 PMC9028248

[84] Fu H Tang B Lang J Du Y Cao B Jin L. High-fat diet promotes macrophage-mediated hepatic inflammation and aggravates diethylnitrosamine-induced hepatocarcinogenesis in mice. Front Nutr (2020) 7:585306. doi: 10.3389/fnut.2020.585306 33304918 PMC7701255

[85] Chen CL Tsukamoto H Liu JC Kashiwabara C Feldman D Sher L. Reciprocal regulation by TLR4 and TGF-beta in tumor-initiating stem-like cells. J Clin Invest (2013) 123(7):2832–49. doi: 10.1172/JCI65859 PMC3696549 23921128

[86] Uthaya Kumar DB Chen CL Liu JC Feldman DE Sher LS French S. TLR4 signaling *via* NANOG cooperates with STAT3 to activate Twist1 and promote formation of tumor-initiating stem-like cells in livers of mice. Gastroenterology (2016) 150(3):707–19. doi: 10.1053/j.gastro.2015.11.002 PMC4766021 26582088

[87] Ruiz de Galarreta M Bresnahan E Molina-Sanchez P Lindblad KE Maier B Sia D. Beta-catenin activation promotes immune escape and resistance to anti-PD-1 therapy in hepatocellular carcinoma. Cancer Discovery (2019) 9(8):1124–41. doi: 10.1158/2159-8290.CD-19-0074 PMC6677618 31186238

[88] Chen J Debebe A Zeng N Kopp J He L Sander M. Transformation of SOX9(+) cells by pten deletion synergizes with steatotic liver injury to drive development of hepatocellular and cholangiocarcinoma. Sci Rep (2021) 11(1):11823. doi: 10.1038/s41598-021-90958-1 34083580 PMC8175600

[89] Jiang A Okabe H Popovic B Preziosi ME Pradhan-Sundd T Poddar M. Loss of wnt secretion by macrophages promotes hepatobiliary injury after administration of 3,5-Diethoxycarbonyl-1, 4-dihydrocollidine diet. Am J Pathol (2019) 189(3):590–603. doi: 10.1016/j.ajpath.2018.11.010 PMC6436111 30610845

[90] Miura K Ishioka M Minami S Horie Y Ohshima S Goto T. Toll-like receptor 4 on macrophage promotes the development of steatohepatitis-related hepatocellular carcinoma in mice. J Biol Chem (2016) 291(22):11504–17. doi: 10.1074/jbc.M115.709048 PMC4882422 27022031

[91] Viebahn CS Benseler V Holz LE Elsegood CL Vo M Bertolino P. Invading macrophages play a major role in the liver progenitor cell response to chronic liver injury. J Hepatol (2010) 53(3):500–7. doi: 10.1016/j.jhep.2010.04.010 20561705

[92] Roggin KK Papa EF Kurkchubasche AG Tracy TF Jr. Kupffer cell inactivation delays repair in a rat model of reversible biliary obstruction. J Surg Res (2000) 90(2):166–73. doi: 10.1006/jsre.2000.5879 10792959

[93] Diehl AM Rai R. Review: regulation of liver regeneration by pro-inflammatory cytokines. J Gastroenterol Hepatol (1996) 11(5):466–70. doi: 10.1111/j.1440-1746.1996.tb00292.x 8743919

[94] Miura A Hosono T Seki T. Macrophage potentiates the recovery of liver zonation and metabolic function after acute liver injury. Sci Rep (2021) 11(1):9730. doi: 10.1038/s41598-021-88989-9 33958644 PMC8102573

[95] Li XF Chen C Xiang DM Qu L Sun W Lu XY. Chronic inflammation-elicited liver progenitor cell conversion to liver cancer stem cell with clinical significance. Hepatol (Baltimore Md. (2017) 66(6):1934–51. doi: 10.1002/hep.29372 28714104

[96] Yang X Shao C Duan L Hou X Huang Y Gao L. Oncostatin m promotes hepatic progenitor cell activation and hepatocarcinogenesis *via* macrophage-derived tumor necrosis factor-alpha. Cancer Lett (2021) 517:46–54. doi: 10.1016/j.canlet.2021.05.039 34102284

[97] He G Dhar D Nakagawa H Font-Burgada J Ogata H Jiang Y. Identification of liver cancer progenitors whose malignant progression depends on autocrine IL-6 signaling. Cell (2013) 155(2):384–96. doi: 10.1016/j.cell.2013.09.031 PMC4015514 24120137

[98] Passman AM Strauss RP McSpadden SB Finch-Edmondson M Andrewartha N Woo KH. Maraviroc prevents HCC development by suppressing macrophages and the liver progenitor cell response in a murine chronic liver disease model. Cancers (Basel) (2021) 13(19):4935. doi: 10.3390/cancers13194935 34638423 PMC8508380

[99] Lee KJ Kim MY Han YH. Roles of heterogenous hepatic macrophages in the progression of liver diseases. BMB Rep (2022) 55(4):166–74. doi: 10.5483/BMBRep.2022.55.4.022 PMC9058466 35321784

[100] Zai W Chen W Liu H Ju D. Therapeutic opportunities of IL-22 in non-alcoholic fatty liver disease: From molecular mechanisms to clinical applications. Biomedicines (2021) 9(12). doi: 10.3390/biomedicines9121912 PMC8698419 34944732

[101] Shriki A Lanton T Sonnenblick A Levkovitch-Siany O Eidelshtein D Abramovitch R. Multiple roles of IL6 in hepatic injury, steatosis, and senescence aggregate to suppress tumorigenesis. Cancer Res (2021) 81(18):4766–77. doi: 10.1158/0008-5472.CAN-21-0321 34117031

[102] Eso Y Takai A Matsumoto T Inuzuka T Horie T Ono K. MSH2 dysregulation is triggered by proinflammatory cytokine stimulation and is associated with liver cancer development. Cancer Res (2016) 76(15):4383–93. doi: 10.1158/0008-5472.CAN-15-2926 27261510

[103] Liaskou E Zimmermann HW Li KK Oo YH Suresh S Stamataki Z. Monocyte subsets in human liver disease show distinct phenotypic and functional characteristics. Hepatol (Baltimore Md. (2013) 57(1):385–98. doi: 10.1002/hep.26016 PMC4194426 22911542

[104] Petrasek J Bala S Csak T Lippai D Kodys K Menashy V. IL-1 receptor antagonist ameliorates inflammasome-dependent alcoholic steatohepatitis in mice. J Clin Invest (2012) 122(10):3476–89. doi: 10.1172/JCI60777 PMC3461900 22945633

[105] McVicker BL Tuma DJ Kharbanda KK Kubik JL Casey CA. Effect of chronic ethanol administration on the *in vitro* production of proinflammatory cytokines by rat kupffer cells in the presence of apoptotic cells. Alcohol Clin Exp Res (2007) 31(1):122–9. doi: 10.1111/j.1530-0277.2006.00270.x 17207110

[106] Bonar E Dubin A Bierczynska-Krzysik A Noga M Silberring J Stalinska K. Identification of major cellular proteins synthesized in response to interleukin-1 and interleukin-6 in human hepatoma HepG2 cells. Cytokine (2006) 33(2):111–7. doi: 10.1016/j.cyto.2005.12.011 16483792

[107] Wan J Benkdane M Teixeira-Clerc F Bonnafous S Louvet A Lafdil F. M2 kupffer cells promote M1 kupffer cell apoptosis: a protective mechanism against alcoholic and nonalcoholic fatty liver disease. Hepatol (Baltimore Md. (2014) 59(1):130–42. doi: 10.1002/hep.26607 23832548

[108] Gao B. Hepatoprotective and anti-inflammatory cytokines in alcoholic liver disease. J Gastroenterol Hepatol (2012) 27 Suppl 2:89–93. doi: 10.1111/j.1440-1746.2011.07003.x PMC3281557 22320924

[109] Nakagawa H Umemura A Taniguchi K Font-Burgada J Dhar D Ogata H. ER stress cooperates with hypernutrition to trigger TNF-dependent spontaneous HCC development. Cancer Cell (2014) 26(3):331–43. doi: 10.1016/j.ccr.2014.07.001 PMC4165611 25132496

[110] Cubero FJ Singh A Borkham-Kamphorst E Nevzorova YA Al Masaoudi M Haas U. TNFR1 determines progression of chronic liver injury in the IKKgamma/Nemo genetic model. Cell Death Differ (2013) 20(11):1580–92. doi: 10.1038/cdd.2013.112 PMC3792433 23933814

[111] Jing Y Sun K Liu W Sheng D Zhao S Gao L. Tumor necrosis factor-alpha promotes hepatocellular carcinogenesis through the activation of hepatic progenitor cells. Cancer Lett (2018) 434:22–32. doi: 10.1016/j.canlet.2018.07.001 29981431

[112] Yuan D Huang S Berger E Liu L Gross N Heinzmann F. Kupffer cell-derived tnf triggers cholangiocellular tumorigenesis through JNK due to chronic mitochondrial dysfunction and ROS. Cancer Cell (2017) 31(6):771–89.e6. doi: 10.1016/j.ccell.2017.05.006 PMC7909318 28609656

[113] Wieckowska A Papouchado BG Li Z Lopez R Zein NN Feldstein AE. Increased hepatic and circulating interleukin-6 levels in human nonalcoholic steatohepatitis. Am J Gastroenterol (2008) 103(6):1372–9. doi: 10.1111/j.1572-0241.2007.01774.x 18510618

[114] Schmidt-Arras D Rose-John S. IL-6 pathway in the liver: From physiopathology to therapy. J Hepatol (2016) 64(6):1403–15. doi: 10.1016/j.jhep.2016.02.004 26867490

[115] Bergmann J Muller M Baumann N Reichert M Heneweer C Bolik J. IL-6 trans-signaling is essential for the development of hepatocellular carcinoma in mice. Hepatol (Baltimore Md. (2017) 65(1):89–103. doi: 10.1002/hep.28874 27770462

[116] Moran DM Mattocks MA Cahill PA Koniaris LG McKillop IH. Interleukin-6 mediates G(0)/G(1) growth arrest in hepatocellular carcinoma through a STAT 3-dependent pathway. J Surg Res (2008) 147(1):23–33. doi: 10.1016/j.jss.2007.04.022 PMC2587231 17574577

[117] Prieto J. Inflammation, HCC and sex: IL-6 in the centre of the triangle. J Hepatol (2008) 48(2):380–1. doi: 10.1016/j.jhep.2007.11.007 18093689

[118] Zhou TY Zhou YL Qian MJ Fang YZ Ye S Xin WX. Interleukin-6 induced by YAP in hepatocellular carcinoma cells recruits tumor-associated macrophages. J Pharmacol Sci (2018) 138(2):89–95. doi: 10.1016/j.jphs.2018.07.013 30340922

[119] Kroy DC Beraza N Tschaharganeh DF Sander LE Erschfeld S Giebeler A. Lack of interleukin-6/glycoprotein 130/signal transducers and activators of transcription-3 signaling in hepatocytes predisposes to liver steatosis and injury in mice. Hepatol (Baltimore Md. (2010) 51(2):463–73. doi: 10.1002/hep.23322 19918973

[120] Wu J Li J Salcedo R Mivechi NF Trinchieri G Horuzsko A. The proinflammatory myeloid cell receptor TREM-1 controls kupffer cell activation and development of hepatocellular carcinoma. Cancer Res (2012) 72(16):3977–86. doi: 10.1158/0008-5472.CAN-12-0938 PMC3694446 22719066

[121] Bi H Zhang Y Wang S Fang W He W Yin L. Interleukin-8 promotes cell migration *via* CXCR1 and CXCR2 in liver cancer. Oncol Lett (2019) 18(4):4176–84. doi: 10.3892/ol.2019.10735 PMC6732969 31516616

[122] Khazali AS Clark AM Wells A. Inflammatory cytokine IL-8/CXCL8 promotes tumour escape from hepatocyte-induced dormancy. Br J Cancer (2018) 118(4):566–76. doi: 10.1038/bjc.2017.414 PMC5830588 29169181

[123] Nagata T McKinley L Peschon JJ Alcorn JF Aujla SJ Kolls JK. Requirement of IL-17RA in con a induced hepatitis and negative regulation of IL-17 production in mouse T cells. J Immunol (2008) 181(11):7473–9. doi: 10.4049/jimmunol.181.11.7473 19017936

[124] Beringer A Miossec P. IL-17 and IL-17-producing cells and liver diseases, with focus on autoimmune liver diseases. Autoimmun Rev (2018) 17(12):1176–85. doi: 10.1016/j.autrev.2018.06.008 30321671

[125] Eguchi A Yan R Pan SQ Wu R Kim J Chen Y. Comprehensive characterization of hepatocyte-derived extracellular vesicles identifies direct miRNA-based regulation of hepatic stellate cells and DAMP-based hepatic macrophage IL-1beta and IL-17 upregulation in alcoholic hepatitis mice. J Mol Med (Berl) (2020) 98(7):1021–34. doi: 10.1007/s00109-020-01926-7 PMC7810220 32556367

[126] Meng F Wang K Aoyama T Grivennikov SI Paik Y Scholten D. Interleukin-17 signaling in inflammatory, kupffer cells, and hepatic stellate cells exacerbates liver fibrosis in mice. Gastroenterology (2012) 143(3):765–76 e3. doi: 10.1053/j.gastro.2012.05.049 PMC3635475 22687286

[127] Ma HY Yamamoto G Xu J Liu X Karin D Kim JY. IL-17 signaling in steatotic hepatocytes and macrophages promotes hepatocellular carcinoma in alcohol-related liver disease. J Hepatol (2020) 72(5):946–59. doi: 10.1016/j.jhep.2019.12.016 PMC7167339 31899206

[128] Shrivastava S Mukherjee A Ray R Ray RB. Hepatitis c virus induces interleukin-1beta (IL-1beta)/IL-18 in circulatory and resident liver macrophages. J Virol (2013) 87(22):12284–90. doi: 10.1128/JVI.01962-13 PMC3807883 24006444

[129] Kimura K Sekiguchi S Hayashi S Hayashi Y Hishima T Nagaki M. Role of interleukin-18 in intrahepatic inflammatory cell recruitment in acute liver injury. J Leukoc Biol (2011) 89(3):433–42. doi: 10.1189/jlb.0710412 21106643

[130] Nguyen NT Umbaugh DS Sanchez-Guerrero G Ramachandran A Jaeschke H. Kupffer cells regulate liver recovery through induction of chemokine receptor CXCR2 on hepatocytes after acetaminophen overdose in mice. Arch Toxicol (2022) 96(1):305–20. doi: 10.1007/s00204-021-03183-0 PMC8762790 34724096

[131] Wehr A Baeck C Heymann F Niemietz PM Hammerich L Martin C. Chemokine receptor CXCR6-dependent hepatic NK T cell accumulation promotes inflammation and liver fibrosis. J Immunol (2013) 190(10):5226–36. doi: 10.4049/jimmunol.1202909 23596313

[132] Jiang R Tan Z Deng L Chen Y Xia Y Gao Y. Interleukin-22 promotes human hepatocellular carcinoma by activation of STAT3. Hepatol (Baltimore Md. (2011) 54(3):900–9. doi: 10.1002/hep.24486 21674558

[133] Zhao D Xia L Geng W Xu D Zhong C Zhang J. Metformin suppresses interleukin-22 induced hepatocellular carcinoma by upregulating hippo signaling pathway. J Gastroenterol Hepatol (2021) 36(12):3469–76. doi: 10.1111/jgh.15674 34432321

[134] Saiman Y Friedman SL. The role of chemokines in acute liver injury. Front Physiol (2012) 3:213. doi: 10.3389/fphys.2012.00213 22723782 PMC3379724

[135] Xu L Chen Y Nagashimada M Ni Y Zhuge F Chen G. CC chemokine ligand 3 deficiency ameliorates diet-induced steatohepatitis by regulating liver macrophage recruitment and M1/M2 status in mice. Metabolism (2021) 125:154914. doi: 10.1016/j.metabol.2021.154914 34656648

[136] Chu X Jin Q Chen H Wood GC Petrick A Strodel W. CCL20 is up-regulated in non-alcoholic fatty liver disease fibrosis and is produced by hepatic stellate cells in response to fatty acid loading. J Transl Med (2018) 16(1):108. doi: 10.1186/s12967-018-1490-y 29690903 PMC5937820

[137] Liu LZ Zhang Z Zheng BH Shi Y Duan M Ma LJ. CCL15 recruits suppressive monocytes to facilitate immune escape and disease progression in hepatocellular carcinoma. Hepatol (Baltimore Md. (2019) 69(1):143–59. doi: 10.1002/hep.30134 30070719

[138] Zhao N Dang H Ma L Martin SP Forgues M Ylaya K. Intratumoral γδ T-cell infiltrates, chemokine (C-c motif) ligand 4/Chemokine (C-c motif) ligand 5 protein expression and survival in patients with hepatocellular carcinoma. Hepatol (Baltimore Md. (2021) 73(3):1045–60. doi: 10.1002/hep.31412 PMC9175512 32502310

[139] Morikawa R Nakamoto N Amiya T Chu PS Koda Y Teratani T. Role of CC chemokine receptor 9 in the progression of murine and human non-alcoholic steatohepatitis. J Hepatol (2021) 74(3):511–21. doi: 10.1016/j.jhep.2020.09.033 33038434

[140] Zhang X Han J Man K Li X Du J Chu ES. CXC chemokine receptor 3 promotes steatohepatitis in mice through mediating inflammatory cytokines, macrophages and autophagy. J Hepatol (2016) 64(1):160–70. doi: 10.1016/j.jhep.2015.09.005 26394162

[141] Tacke F Zimmermann HW Trautwein C Schnabl B. CXCL5 plasma levels decrease in patients with chronic liver disease. J Gastroenterol Hepatol (2011) 26(3):523–9. doi: 10.1111/j.1440-1746.2010.06436.x PMC3058722 21332547

[142] Wang T Chen B Meng T Liu Z Wu W. Identification and immunoprofiling of key prognostic genes in the tumor microenvironment of hepatocellular carcinoma. Bioengineered (2021) 12(1):1555–75. doi: 10.1080/21655979.2021.1918538 PMC8806269 33955820

[143] Morales-Ibanez O Bataller R. Platelet-derived chemokines: new targets to treat liver fibrosis. J Hepatol (2011) 54(3):581–3. doi: 10.1016/j.jhep.2010.09.016 21112659

[144] Marra F Tacke F. Roles for chemokines in liver disease. Gastroenterology (2014) 147(3):577–94.e1. doi: 10.1053/j.gastro.2014.06.043 25066692

[145] Zhou Z Xu MJ Cai Y Wang W Jiang JX Varga ZV. Neutrophil-hepatic stellate cell interactions promote fibrosis in experimental steatohepatitis. Cell Mol Gastroenterol Hepatol (2018) 5(3):399–413. doi: 10.1016/j.jcmgh.2018.01.003 PMC5852390 29552626

[146] Noh J-R Kim Y-H Kim D-K Hwang JH Kim K-S Choi D-H. Small heterodimer partner negatively regulates c-X-C motif chemokine ligand 2 in hepatocytes during liver inflammation. Sci Rep (2018) 8(1):15222. doi: 10.1038/s41598-018-33660-z 30323351 PMC6189097

[147] Zimmermann HW Seidler S Gassler N Nattermann J Luedde T Trautwein C. Interleukin-8 is activated in patients with chronic liver diseases and associated with hepatic macrophage accumulation in human liver fibrosis. PloS One (2011) 6(6):e21381. doi: 10.1371/journal.pone.0021381 21731723 PMC3120868

[148] House IG Savas P Lai J Chen AXY Oliver AJ Teo ZL. Macrophage-derived CXCL9 and CXCL10 are required for antitumor immune responses following immune checkpoint blockade. Clin Cancer Res (2020) 26(2):487–504. doi: 10.1158/1078-0432.CCR-19-1868 31636098

[149] Ibrahim SH Hirsova P Tomita K Bronk SF Werneburg NW Harrison SA. Mixed lineage kinase 3 mediates release of c-X-C motif ligand 10-bearing chemotactic extracellular vesicles from lipotoxic hepatocytes. Hepatol (Baltimore Md. (2016) 63(3):731–44. doi: 10.1002/hep.28252 PMC4764421 26406121

[150] Xu C Zhang C Ji J Wang C Yang J Geng B. CD36 deficiency attenuates immune-mediated hepatitis in mice by modulating the proapoptotic effects of CXC chemokine ligand 10. Hepatol (Baltimore Md. (2018) 67(5):1943–55. doi: 10.1002/hep.29716 29220536

[151] Gerhard GS Legendre C Still CD Chu X Petrick A DiStefano JK. Transcriptomic profiling of obesity-related nonalcoholic steatohepatitis reveals a core set of fibrosis-specific genes. J Endocr Soc (2018) 2(7):710–26. doi: 10.1210/js.2018-00122 PMC6018672 29978150

[152] Loft A Schmidt SF Caratti G Stifel U Havelund J Sekar R. A macrophage-hepatocyte glucocorticoid receptor axis coordinates fasting ketogenesis. Cell Metab (2022) 34(3):473–86 e9. doi: 10.1016/j.cmet.2022.01.004 35120589

[153] Heymann F Tacke F. Immunology in the liver–from homeostasis to disease. Nat Rev Gastroenterol Hepatol (2016) 13(2):88–110. doi: 10.1038/nrgastro.2015.200 26758786

[154] Faraj TA Stover C Erridge C. Dietary toll-like receptor stimulants promote hepatic inflammation and impair reverse cholesterol transport in mice *via* macrophage-dependent interleukin-1 production. Front Immunol (2019) 10:1404. doi: 10.3389/fimmu.2019.01404 31316501 PMC6611433

[155] Goldstein JL Brown MS. The LDL receptor. Arterioscler Thromb Vasc Biol (2009) 29(4):431–8. doi: 10.1161/ATVBAHA.108.179564 PMC2740366 19299327

[156] Fisher JE McKenzie TJ Lillegard JB Yu Y Juskewitch JE Nedredal GI. Role of kupffer cells and toll-like receptor 4 in acetaminophen-induced acute liver failure. J Surg Res (2013) 180(1):147–55. doi: 10.1016/j.jss.2012.11.051 PMC3578072 23260383

[157] Wang H Bloom O Zhang M Vishnubhakat JM Ombrellino M Che J. HMG-1 as a late mediator of endotoxin lethality in mice. Science (1999) 285(5425):248–51. doi: 10.1126/science.285.5425.248 10398600

[158] Li L Chen L Hu L Liu Y Sun HY Tang J. Nuclear factor high-mobility group box1 mediating the activation of toll-like receptor 4 signaling in hepatocytes in the early stage of nonalcoholic fatty liver disease in mice. Hepatol (Baltimore Md. (2011) 54(5):1620–30. doi: 10.1002/hep.24552 21809356

[159] Ioannou GN Subramanian S Chait A Haigh WG Yeh MM Farrell GC. Cholesterol crystallization within hepatocyte lipid droplets and its role in murine NASH. J Lipid Res (2017) 58(6):1067–79. doi: 10.1194/jlr.M072454 PMC5456359 28404639

[160] Lara-Guzman OJ Gil-Izquierdo A Medina S Osorio E Alvarez-Quintero R Zuluaga N. Oxidized LDL triggers changes in oxidative stress and inflammatory biomarkers in human macrophages. Redox Biol (2018) 15:1–11. doi: 10.1016/j.redox.2017.11.017 PMC5723280 29195136

[161] Tang SP Mao XL Chen YH Yan LL Ye LP Li SW. Reactive oxygen species induce fatty liver and ischemia-reperfusion injury by promoting inflammation and cell death. Front Immunol (2022) 13:870239. doi: 10.3389/fimmu.2022.870239 35572532 PMC9098816

[162] Bieghs V Verheyen F van Gorp PJ Hendrikx T Wouters K Lutjohann D. Internalization of modified lipids by CD36 and SR-a leads to hepatic inflammation and lysosomal cholesterol storage in kupffer cells. PloS One (2012) 7(3):e34378. doi: 10.1371/journal.pone.0034378 22470565 PMC3314620

[163] Rivera K Quinones V Amigo L Santander N Salas-Perez F Xavier A. Lipoprotein receptor SR-B1 deficiency enhances adipose tissue inflammation and reduces susceptibility to hepatic steatosis during diet-induced obesity in mice. Biochim Biophys Acta Mol Cell Biol Lipids (2021) 1866(6):158909. doi: 10.1016/j.bbalip.2021.158909 33631309

[164] Gonzalez de la Aleja A Herrero C Torres-Torresano M de la Rosa JV Alonso B Capa-Sardon E. Activation of LXR nuclear receptors impairs the anti-inflammatory gene and functional profile of m-CSF-Dependent human monocyte-derived macrophages. Front Immunol (2022) 13:835478. doi: 10.3389/fimmu.2022.835478 35280993 PMC8907538

[165] Laffitte BA Repa JJ Joseph SB Wilpitz DC Kast HR Mangelsdorf DJ. LXRs control lipid-inducible expression of the apolipoprotein e gene in macrophages and adipocytes. Proc Natl Acad Sci USA (2001) 98(2):507–12. doi: 10.1073/pnas.98.2.507 PMC14617 11149950

[166] Moore KJ Rosen ED Fitzgerald ML Randow F Andersson LP Altshuler D. The role of PPAR-gamma in macrophage differentiation and cholesterol uptake. Nat Med (2001) 7(1):41–7. doi: 10.1038/83328 11135614

[167] Malerod L Sporstol M Juvet LK Mousavi A Gjoen T Berg T. Hepatic scavenger receptor class b, type I is stimulated by peroxisome proliferator-activated receptor gamma and hepatocyte nuclear factor 4alpha. Biochem Biophys Res Commun (2003) 305(3):557–65. doi: 10.1016/S0006-291X(03)00819-2 12763030

[168] Feng J Han J Pearce SF Silverstein RL Gotto AM Jr. Hajjar DP. Induction of CD36 expression by oxidized LDL and IL-4 by a common signaling pathway dependent on protein kinase c and PPAR-gamma. J Lipid Res (2000) 41(5):688–96. doi: 10.1016/S0022-2275(20)32377-4 10787429

[169] Song Z Xia H Yang L Wang S Sun G. Lowering the n-6/n-3 PUFAs ratio inhibits the formation of THP-1 macrophage-derived foam cell. Lipids Health Dis (2018) 17(1):125. doi: 10.1186/s12944-018-0772-y 29801502 PMC5970467

[170] Nagy L Tontonoz P Alvarez JG Chen H Evans RM. Oxidized LDL regulates macrophage gene expression through ligand activation of PPARgamma. Cell (1998) 93(2):229–40. doi: 10.1016/S0092-8674(00)81574-3 9568715

[171] Ampomah PB Cai B Sukka SR Gerlach BD Yurdagul A Jr. Wang X. Macrophages use apoptotic cell-derived methionine and DNMT3A during efferocytosis to promote tissue resolution. Nat Metab (2022) 4(4):444–57. doi: 10.1038/s42255-022-00551-7 PMC9050866 35361955

[172] Xu M Wang X Li Y Geng X Jia X Zhang L. Arachidonic acid metabolism controls macrophage alternative activation through regulating oxidative phosphorylation in PPARgamma dependent manner. Front Immunol (2021) 12:618501. doi: 10.3389/fimmu.2021.618501 34149684 PMC8211451

[173] Bujold K Rhainds D Jossart C Febbraio M Marleau S Ong H. CD36-mediated cholesterol efflux is associated with PPARgamma activation *via* a MAPK-dependent COX-2 pathway in macrophages. Cardiovasc Res (2009) 83(3):457–64. doi: 10.1093/cvr/cvp118 19377069

[174] Wojcik M Ramadori P Blaschke M Sultan S Khan S Malik IA. Immunodetection of cyclooxygenase-2 (COX-2) is restricted to tissue macrophages in normal rat liver and to recruited mononuclear phagocytes in liver injury and cholangiocarcinoma. Histochem Cell Biol (2012) 137(2):217–33. doi: 10.1007/s00418-011-0889-9 PMC3262142 22131058

[175] Diaz-Gandarilla JA Osorio-Trujillo C Hernandez-Ramirez VI Talamas-Rohana P. PPAR activation induces M1 macrophage polarization *via* cPLA(2)-COX-2 inhibition, activating ROS production against leishmania mexicana. BioMed Res Int (2013) 2013:215283. doi: 10.1155/2013/215283 23555077 PMC3600276

[176] Tao HC Chen KX Wang X Chen B Zhao WO Zheng Y. CD47 deficiency in mice exacerbates chronic fatty diet-induced steatohepatitis through its role in regulating hepatic inflammation and lipid metabolism. Front Immunol (2020) 11:148. doi: 10.3389/fimmu.2020.00148 32158445 PMC7052326

[177] Bi J Sun K Wu H Chen X Tang H Mao J. PPARgamma alleviated hepatocyte steatosis through reducing SOCS3 by inhibiting JAK2/STAT3 pathway. Biochem Biophys Res Commun (2018) 498(4):1037–44. doi: 10.1016/j.bbrc.2018.03.110 29550470

[178] Koundouros N Poulogiannis G. Reprogramming of fatty acid metabolism in cancer. Br J Cancer (2020) 122(1):4–22. doi: 10.1038/s41416-019-0650-z PMC6964678 31819192

[179] Fishbein A Wang W Yang H Yang J Hallisey VM Deng J. Resolution of eicosanoid/cytokine storm prevents carcinogen and inflammation-initiated hepatocellular cancer progression. Proc Natl Acad Sci USA (2020) 117(35):21576–87. doi: 10.1073/pnas.2007412117 PMC7474612 32801214

[180] Hao H Liu M Wu P Cai L Tang K Yi P. Lipoxin A4 and its analog suppress hepatocellular carcinoma *via* remodeling tumor microenvironment. Cancer Lett (2011) 309(1):85–94. doi: 10.1016/j.canlet.2011.05.020 21683517

[181] Xu YJ Zheng Z Cao C Li J Liu Y. Bioanalytical insights into the association between eicosanoids and pathogenesis of hepatocellular carcinoma. Cancer Metastasis Rev (2018) 37(2-3):269–77. doi: 10.1007/s10555-018-9747-8 29934821

[182] Maciejewska D Drozd A Skonieczna-Zydecka K Skorka-Majewicz M Dec K Jakubczyk K. Eicosanoids in nonalcoholic fatty liver disease (NAFLD) progression. do serum eicosanoids profile correspond with liver eicosanoids content during NAFLD development and progression? Molecules (2020) 25(9):2026. doi: 10.3390/molecules25092026 PMC7248881 32349225

[183] Pandey V Sultan M Kashofer K Ralser M Amstislavskiy V Starmann J. Comparative analysis and modeling of the severity of steatohepatitis in DDC-treated mouse strains. PloS One (2014) 9(10):e111006. doi: 10.1371/journal.pone.0111006 25347188 PMC4210132

[184] Qian Y Fan JG. Obesity, fatty liver and liver cancer. Hepatobiliary Pancreat Dis Int (2005) 4(2):173–7. 15908310

[185] de Oliveira S Houseright RA Graves AL Golenberg N Korte BG Miskolci V. Metformin modulates innate immune-mediated inflammation and early progression of NAFLD-associated hepatocellular carcinoma in zebrafish. J Hepatol (2019) 70(4):710–21. doi: 10.1016/j.jhep.2018.11.034 PMC6436385 30572006

[186] Horiuchi T Sakata N Narumi Y Kimura T Hayashi T Nagano K. Metformin directly binds the alarmin HMGB1 and inhibits its proinflammatory activity. J Biol Chem (2017) 292(20):8436–46. doi: 10.1074/jbc.M116.769380 PMC5437248 28373282

[187] Marrache MK Rockey DC. Statins for treatment of chronic liver disease. Curr Opin Gastroenterol (2021) 37(3):200–7. doi: 10.1097/MOG.0000000000000716 PMC8691140 33654016

[188] Zou B Odden MC Nguyen MH. Statin use and reduced hepatocellular carcinoma risk in patients with nonalcoholic fatty liver disease. Clin Gastroenterol Hepatol (2022) S1542–3565(22):00137–9. doi: 10.1016/j.cgh.2022.01.057 35158055

[189] Hajifathalian K Tafesh Z Rosenblatt R Kumar S Homan EA Sharaiha RZ. Effect of statin use on cancer-related mortality in nonalcoholic fatty liver disease: A prospective united states cohort study. J Clin Gastroenterol (2022) 56(2):173–80. doi: 10.1097/MCG.0000000000001503 33606428

[190] Pinyopornpanish K Al-Yaman W Butler RS Carey W McCullough A Romero-Marrero C. Chemopreventive effect of statin on hepatocellular carcinoma in patients with nonalcoholic steatohepatitis cirrhosis. Am J Gastroenterol (2021) 116(11):2258–69. doi: 10.14309/ajg.0000000000001347 34212895

[191] Dehnavi S Kiani A Sadeghi M Biregani AF Banach M Atkin SL. Targeting AMPK by statins: A potential therapeutic approach. Drugs (2021) 81(8):923–33. doi: 10.1007/s40265-021-01510-4 PMC8144155 33939118

[192] Gao J Xiong R Xiong D Zhao W Zhang S Yin T. The adenosine monophosphate (AMP) analog, 5-Aminoimidazole-4-Carboxamide ribonucleotide (AICAR) inhibits hepatosteatosis and liver tumorigenesis in a high-fat diet murine model treated with diethylnitrosamine (DEN). Med Sci Monit (2018) 24:8533–43. doi: 10.12659/MSM.910544 PMC6278641 30474622

[193] Wang MD Wang NY Zhang HL Sun LY Xu QR Liang L. Fatty acid transport protein-5 (FATP5) deficiency enhances hepatocellular carcinoma progression and metastasis by reprogramming cellular energy metabolism and regulating the AMPK-mTOR signaling pathway. Oncogenesis (2021) 10(11):74. doi: 10.1038/s41389-021-00364-5 34772914 PMC8589992

[194] Jia C Medina V Liu C He L Qian D Taojian T. Crosstalk of LKB1- and PTEN-regulated signals in liver morphogenesis and tumor development. Hepatol Commun (2017) 1(2):153–67. doi: 10.1002/hep4.1027 PMC5687583 29152604

[195] Jian C Fu J Cheng X Shen LJ Ji YX Wang X. Low-dose sorafenib acts as a mitochondrial uncoupler and ameliorates nonalcoholic steatohepatitis. Cell Metab (2020) 31(5):892–908.e11. 10.1016/j.cmet.2020.04.011 PMC9375823 32375062

[196] Chen CY Li Y Zeng N He L Zhang X Tu T. Inhibition of estrogen-related receptor alpha blocks liver steatosis and steatohepatitis and attenuates triglyceride biosynthesis. Am J Pathol (2021) 191(7):1240–54. doi: 10.1016/j.ajpath.2021.04.007 PMC8261472 33894178

[197] Xia H Dufour CR Giguere V. ERRalpha as a bridge between transcription and function: Role in liver metabolism and disease. Front Endocrinol (Lausanne) (2019) 10:206. doi: 10.3389/fendo.2019.00206 31024446 PMC6459935

[198] Liao H Chen W Dai Y Richardson JJ Guo J Yuan K. Expression of programmed cell death-ligands in hepatocellular carcinoma: Correlation with immune microenvironment and survival outcomes. Front Oncol (2019) 9:883. doi: 10.3389/fonc.2019.00883 31572677 PMC6749030

[199] Kuang DM Zhao Q Peng C Xu J Zhang JP Wu C. Activated monocytes in peritumoral stroma of hepatocellular carcinoma foster immune privilege and disease progression through PD-L1. J Exp Med (2009) 206(6):1327–37. doi: 10.1084/jem.20082173 PMC2715058 19451266

[200] Yu J Green MD Li S Sun Y Journey SN Choi JE. Liver metastasis restrains immunotherapy efficacy *via* macrophage-mediated T cell elimination. Nat Med (2021) 27(1):152–64. doi: 10.1038/s41591-020-1131-x PMC8095049 33398162

[201] Jacques A Bleau C Martin JP Lamontagne L. Intrahepatic endothelial and kupffer cells involved in immunosuppressive cytokines and natural killer (NK)/NK T cell disorders in viral acute hepatitis. Clin Exp Immunol (2008) 152(2):298–310. doi: 10.1111/j.1365-2249.2008.03628.x PMC2384105 18336588

[202] Knolle PA Uhrig A Hegenbarth S Loser E Schmitt E Gerken G. IL-10 down-regulates T cell activation by antigen-presenting liver sinusoidal endothelial cells through decreased antigen uptake *via* the mannose receptor and lowered surface expression of accessory molecules. Clin Exp Immunol (1998) 114(3):427–33. 10.1046/j.1365-2249.1998.00713.x PMC1905120 9844054

[203] You Q Cheng L Kedl RM Ju C. Mechanism of T cell tolerance induction by murine hepatic kupffer cells. Hepatol (Baltimore Md. (2008) 48(3):978–90. doi: 10.1002/hep.22395 PMC2600585 18712788

[204] Pfister D Nunez NG Pinyol R Govaere O Pinter M Szydlowska M. NASH limits anti-tumour surveillance in immunotherapy-treated HCC. Nature (2021) 592(7854):450–6. doi: 10.1038/s41586-021-03362-0 PMC8046670 33762733

[205] McLaren JE Michael DR Guschina IA Harwood JL Ramji DP. Eicosapentaenoic acid and docosahexaenoic acid regulate modified LDL uptake and macropinocytosis in human macrophages. Lipids (2011) 46(11):1053–61. doi: 10.1007/s11745-011-3598-1 21822944

[206] Pietsch A Weber C Goretzki M Weber PC Lorenz RL. N-3 but not n-6 fatty acids reduce the expression of the combined adhesion and scavenger receptor CD36 in human monocytic cells. Cell Biochem Funct (1995) 13(3):211–6. doi: 10.1002/cbf.290130312 7554100

[207] Liebig M Dannenberger D Vollmar B Abshagen K. Endogenously increased n-3 PUFA levels in fat-1 transgenic mice do not protect from non-alcoholic steatohepatitis. Hepatobiliary Surg Nutr (2019) 8(5):447–58. doi: 10.21037/hbsn.2019.04.03 PMC6791993 31673534

[208] Behari J Yeh TH Krauland L Otruba W Cieply B Hauth B. Liver-specific beta-catenin knockout mice exhibit defective bile acid and cholesterol homeostasis and increased susceptibility to diet-induced steatohepatitis. Am J Pathol (2010) 176(2):744–53. doi: 10.2353/ajpath.2010.090667 PMC2808081 20019186

[209] NCI. SEER cancer statistics review (2004). Available at: http://seercancergov/csr/1975_2003/.

[210] WHO. Cancer epidemiology data base (2002). Available at: http://www-depiarcfr/.

[211] Wilson JF. Liver cancer on the rise. Ann Internal Med (2005) 142(12 Pt 1):1029–32. doi: 10.7326/0003-4819-142-12_Part_1-200506210-00024 15968025

